# Chemoprevention of Barrett’s Esophagus: a Systematic Review and Comprehensive Assessment of Bias

**DOI:** 10.1093/dote/doaf062

**Published:** 2025-08-06

**Authors:** Mie Thu Ko, Agha Rizwanullah, Zain Jafri, Adriel Fung, Leo Alexandre

**Affiliations:** Norwich Medical School, University of East Anglia, Norwich, UK; Department of Gastroenterology, Norfolk and Norwich University Hospital NHS Foundation Trust, Norwich, UK; Norwich Medical School, University of East Anglia, Norwich, UK; Department of Gastroenterology, Norfolk and Norwich University Hospital NHS Foundation Trust, Norwich, UK; Norwich Medical School, University of East Anglia, Norwich, UK; Norwich Medical School, University of East Anglia, Norwich, UK; Department of Gastroenterology, Norfolk and Norwich University Hospital NHS Foundation Trust, Norwich, UK; Norwich Medical School, University of East Anglia, Norwich, UK; Department of Gastroenterology, Norfolk and Norwich University Hospital NHS Foundation Trust, Norwich, UK

**Keywords:** Barrett’s esophagus, esophageal adenocarcinoma, high-grade dysplasia, chemoprevention

## Abstract

Chemoprevention of Barrett’s esophagus (BE) represents an opportunity to reduce the burden of esophageal adenocarcinoma (EAC). We conducted a systematic review and meta-analysis to evaluate the assumed causal association between proton-pump inhibitors (PPIs), aspirin and statins, and BE progression, and undertook a comprehensive risk of bias (RoB) assessment. The protocol was prospectively registered (PROSPERO ID: CRD42024532338). Sixteen observational studies and one randomized controlled trial were identified. PPIs and statins were associated with a 54% (adjusted OR 0.46; 95% CI 0.25–0.86; *P* = 0.02) and 47% (adjusted OR 0.53; 95% CI 0.37–0.74; *P* < 0.001) reduced odds of progression, and aspirin use was not significantly associated (adjusted OR 0.84; 95% CI 0.65–1.08; *P* = 0.17). Among observational studies, 6 were at critical RoB and 10 were at serious RoB. The only trial included was at low RoB and reported no significant associations for aspirin and PPI comparisons and high-grade dysplasia (HGD)/EAC. The Grading of Recommendations, Assessment, Development and Evaluations certainty of evidence was very low. All observational studies were at serious or critical RoB. Trial evidence was at low RoB and did not demonstrate any significant differences between aspirin and PPI comparisons for the outcome of HGD/EAC. Given the very low certainty of evidence, there is little rationale to recommend these medications for chemoprevention in BE.

## INTRODUCTION

Barrett’s esophagus (BE) is the precursor lesion to esophageal adenocarcinoma (EAC), an aggressive cancer with a poor prognosis, particularly when diagnosed in symptomatic patients.^1^ BE is associated with a 30-fold increase in the incidence of EAC.^2^ Patients with BE are at a 13-fold relative increase in death from EAC compared to the general population.^3^ The process of carcinogenesis follows a well-characterized metaplasia–dysplasia–adenocarcinoma sequence, where non-dysplastic BE (NDBE) progresses to dysplasia (low-grade dysplasia ([LGD]) then high-grade dysplasia [HGD]), to intramucosal adenocarcinoma and then invasive disease.^4^ The overall annual risk of malignant progression of NDBE has been estimated at 0.33%.^5^ The population prevalence of BE may substantially rise in the future with the emergence of effective screening tools, such as the Cytosponge-trefoil factor 3 test.^6^ Prevention of malignant progression could reduce the EAC-related burden to healthcare systems.

Endoscopic surveillance of BE is widely practiced internationally to prevent invasive EAC or aid early cancer diagnosis; however, there is a lack of direct evidence from randomized trials demonstrating its efficacy and there are notable tradeoffs.^7^ Diagnostic accuracy for dysplasia is imperfect, endoscopic surveillance is invasive and expensive, it has a substantial carbon footprint, patients often require intravenous sedation, and endoscopy is associated with uncommon but serious complications and excess emergency admissions.^8^^,^^9^ Endoscopy services are under substantial pressure with demand exceeding capacity, leading to growing waiting times. There is therefore interest in chemoprevention as a strategy to reduce incident invasive disease, which may in turn potentially reduce the need for surveillance^10^ These candidate chemoprevention medications (CCMs) include proton-pump inhibitors (PPIs), aspirin, and statins.

The recently reported AspECT Trial demonstrated that high-dose PPI in combination with aspirin significantly reduced rates of the composite outcome of all-cause mortality, HGD, or EAC (time ratio [TR] = 1.59; 95% CI 1.14–2.23; *P* = 0.0068).^11^ Notably, this benefit was mainly driven by a reduction in all-cause mortality rather than incident EAC, the latter being the primary aim of Barrett’s chemoprevention. In contrast, previous observational studies have suggested substantial reductions in risk of EAC associated with CCM use.^12–15^ These conflicting findings have led to variable clinical guideline recommendations. The European Society of Gastrointestinal Endoscopy (ESGE) and American College of Gastroenterology Guidelines recommend the use of PPIs as chemoprevention in BE, a weak recommendation based on moderate quality of evidence.^16^^,^^17^ However, the National Institute of Health and Care Excellence (NICE) guidelines on BE management do not recommend the use of PPIs as chemoprevention.^18^

While CCMs might confer chemopreventive effects, alternative explanations for these impressive effect sizes should be sought. Previous systematic reviews have been conducted to determine the association between CCMs and malignant progression of BE, with quality assessment conducted using tools such as the Newcastle-Ottawa Scale (NOS), which suggested that most studies were of moderate to high quality.^19–21^ While the items in the NOS checklist are based on methodologically sound principles, the scale does not comprehensively consider all sources of bias applicable to observational studies of interventions. Newer tools, such as Risk of Bias in Non-randomized studies—of Interventions (ROBINS-I) have since been developed to assess study validity, with a shift in emphasis from methodological quality toward domain-based assessment in which different types of bias are considered in turn to to provide a more comprehensively consider risk of bias (RoB).^22^ Given the clinical importance of accurately assessing the strength of evidence informing guideline recommendations, particularly in the context of discrepancies between different guidelines, rigorous evaluation of study validity is essential. In view of this, the aim of our study is to evaluate the assumed causal association between each CCM and malignant progression of BE, and to perform a comprehensive assessment of RoB using ROBINS-I and Revised Cochrane risk-of-bias tool for randomized trials (RoB 2) tools (applicable to randomized controlled trials).

## METHODS

This systematic review was conducted and reported in accordance with the PRISMA (Preferred Reporting Items for Systematic Reviews and Meta-Analyses) 2020 guidelines.^23^ The protocol was registered on PROSPERO (ID CRD42024532338).

### Information sources and search strategy

We identified relevant articles by searching MEDLINE and EMBASE databases from inception to February 2025 by using the OVID interface (the search strategy is detailed in Supplementary Table S1). We used the following search terms (including related terms) to construct the search strategy: ‘Barrett’s esophagus’, ‘dysplasia’, ‘cancer’, ‘esophageal adenocarcinoma’, ‘progression’, ‘aspirin’, ‘statin’, ‘proton-pump inhibitor’, and ‘chemoprevention’. No language restrictions were applied on the searches. Following this, reference lists of retrieved articles were reviewed to identify any additional studies for inclusion.

### Eligibility criteria

We included observational studies (case–control, cohort, and nested case–control studies) and randomized controlled trials if they met the following eligibility criteria: (1) documented BE (either purely non-dysplastic or a mixed cohort of NDBE and LGD or indefinite for neoplasia or unknown dysplasia status [applicable to studies reliant on diagnostic codes for BE]) at entry for cohort studies or as the control group (who did not progress to HGD/EAC/esophageal cancer (EC) [histological subtype not specified]) in case–control studies; (2) reported outcome of HGD, EAC, or EC; (3) drug exposures and comparisons include PPI, statin, or aspirin use compared with no use, and higher-dose use compared with lower-dose use. We did not put any restrictions on the minimum length of columnar-lined esophagus or whether intestinal metaplasia was required for the definition of BE. Exclusion criteria included: (1) presence of HGD or EAC at baseline; (2) effect sizes or data necessary to calculate effect sizes were not reported. If multiple publications arose from the same population, we included the study with the most relevant and contemporaneous cohort. Two reviewers (AF and ARU) independently screened abstracts and selected full-text articles for inclusion based on the eligibility and exclusion criteria. Discrepancies were resolved through discussion between reviewers.

### Data extraction

Two reviewers (ARU and ZJ) independently extracted data from each selected article for study characteristics (study design, location, setting, recruitment period, definition of BE used, proportion with LGD at baseline, number of patients that progressed to HGD or EAC, definition of progression, follow-up duration, method of ascertainment for medication exposure, the duration of lag period applied, confounders adjusted for); and patient characteristics (age, sex, body mass index [BMI], smoking status, concurrent use of Non-Steroidal Anti-Inflammatory Drugs (NSAIDs), diagnosis of Gastroesophageal Reflux Disease (GERD) prior to BE diagnosis).

### Risk of bias assessment

Evaluation of RoB was completed by using the ROBINS-I tool for observational studies and the RoB 2.^22^^,^^24^ The ROBINS-I tool was developed to assess the RoB in non-randomized studies of interventions (NRSIs) by considering each study as an emulated target trial.^22^ This tool examines the RoB across seven domains: confounding, classification of interventions, selection of participants, deviations from intended interventions, missing data, measurement of outcomes, and selection of reported results. Each domain was considered to have low, moderate, serious, or critical RoB, with the overall RoB determined by the highest risk domain. The RoB assessment related to the effect of assignment to the CCM at baseline, regardless of future adherence (equivalent to intention-to-treat) and not the effect of starting and adhering to treatment (equivalent to the per-protocol effect). The prespecified set of potential confounders used to evaluate bias due to confounding included, as a minimum age, sex, and smoking (see Supplementary Figs S29–S31 which detail the assumed causal relationships and the full lists of confounders). The RoB 2 tool facilitates the assessment of RoB in randomized studies across five domains: randomization process, deviations from the intended interventions, missing outcome data, measurement of the outcome, and selection of the reported result.^24^ Each domain was considered to have ‘low risk of bias’, ‘some concerns’, or ‘high risk of bias’, with the overall study RoB corresponding to the highest risk domain. We used RobVis, an open-access web-based visualization too, to create the figures displaying RoB assessments.^25^ Two reviewers (LA and MTK) completed this assessment independently, and discrepancies were resolved through consensus between reviewers.

The certainty of evidence was evaluated using the GRADE (Grading of Recommendations, Assessment, Development and Evaluations) Framework.^26^ The certainty of evidence for a given outcome was rated as very low, low, moderate, or high using the GRADE Framework. The assessment of the certainty of evidence was completed through consideration of five domains: RoB, inconsistency, indirectness, imprecision, and publication bias.

### Statistical analysis

The primary outcome of interest was progression to HGD/EAC/EC. Adjusted effect sizes, including 95% confidence intervals, were preferentially extracted (or patient counts used for their estimation if required) were extracted from each included study. Since the malignant progression of BE is a relatively rare outcome, effect sizes from either odds-ratio (OR), relative risks (RR), or hazard ratios (HR) would be expected to approximate one another. Meta-analysis was performed using the random effect restricted maximum likelihood (REML) method. *P* values <0.05 were considered statistically significant.

Heterogeneity across studies was evaluated using the inconsistency index (*I*^2^) statistic.^27^  *I*^2^ values of <30%, 30–59%, 60–75%, and ≥75% were classified as low, moderate, substantial, and considerable heterogeneity, respectively. Subgroup analysis was performed based on study design (case–control vs. cohort), study setting (hospital vs. population-based), baseline dysplasia status (non-dysplastic, at most LGD, unknown), study outcome (HGD/EAC/EC), length of BE (presence of columnar lined esophagus [CLE] at least 1 cm vs. not reported), BE definition (biopsy-confirmed vs. coded diagnosis of BE vs. endoscopic appearance only), method of drug exposure ascertainment (medical records/prescription databases, self-reported), and overall RoB assessment. The results of subgroup analyses were considered statistically significant if *P* value for subgroup differences was <0.1.^28^ Publication bias was assessed using a funnel plot and Egger’s regression test if at least ten studies were identified.^29^ All statistical analyses were performed using STATA version 18 (StataCorp LP, College Station, TX, USA).

## RESULTS

### Search and selection of studies

**Fig. 1:**
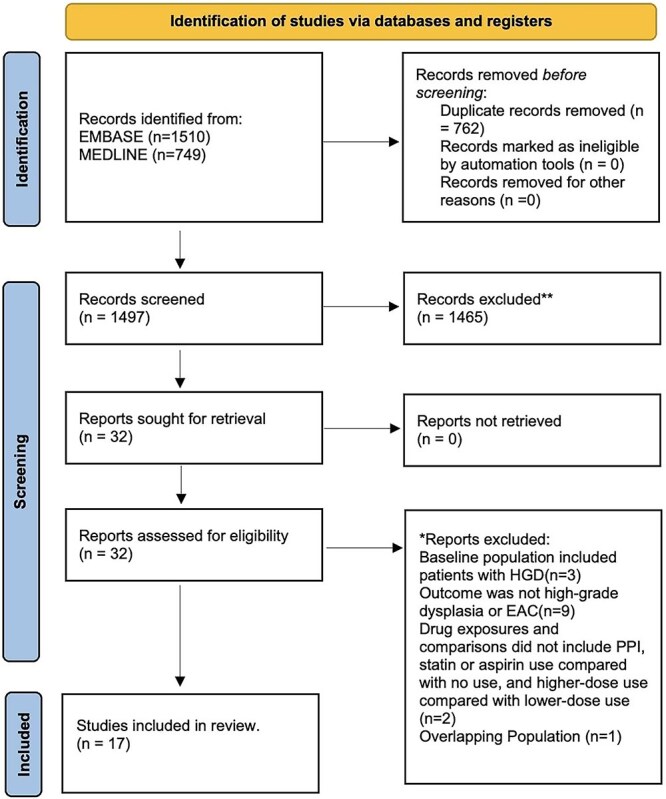
PRISMA flow chart.

Among 2259 articles identified from the literature search, 32 full-text articles were assessed for eligibility, with 17 ultimately selected for inclusion (Fig. 1). Fifteen articles were rejected because the baseline population included patients with HGD (*n* = 3), the reported outcome was not HGD or EAC (*n* = 9), drug exposures and comparisons did not include the use of PPIs, statins, or aspirin, comparing users to non-users, or higher-dose use to lower-dose use (*n* = 2), and one study included overlapping data from a study with a more contemporaneous cohort (*n* = 1).

### Study characteristics

Seventeen studies included 28,141 patients in total, of whom 2444 were diagnosed with HGD/EAC/EC. Study characteristics are presented in Table 1. Nine were from Europe,^12–15^^,^^30–34^ and seven were from the USA.^35–41^ Thirteen studies were cohort studies,^12^^,^^13^^,^^15^^,^^30–32^^,^^34–38^^,^^40^^,^^41^ of which seven were nested case–control studies,^13^^,^^32^^,^^34–37^^,^^40^ three were case–control studies,^14^^,^^33^^,^^39^ and one was a randomized trial with a 2 × 2 factorial design.^11^ Nine were population based,^12^^,^^13^^,^^30^^,^^32^^,^^34–37^^,^^40^ five were multi-center studies,^11^^,^^14^^,^^15^^,^^30^^,^^31^ and three were single-center studies.^33^^,^^38^^,^^41^ BE was defined as the presence of endoscopic appearance of CLE with histological confirmation of intestinal metaplasia in six studies.^14^^,^^15^^,^^31^^,^^33^^,^^38^^,^^41^ The definition of BE was based on clinical codes (e.g. READ or ICD-9 code) in nine studies.^12^^,^^13^^,^^32^^,^^34–37^^,^^39^^,^^40^ Two studies defined BE based on endoscopic appearance only.^11^^,^^30^ Among the 13 cohort and nested case–control studies, baseline dysplasia status was not reported in eight population-based studies due to the absence of detailed histopathology data in the datasets used.^12^^,^^13^^,^^32^^,^^34–37^^,^^40^ In the remaining five studies, the number of NDBE and LGD were reported where the majority of patients (89.3%) were NDBE at baseline.^15^^,^^30^^,^^31^^,^^38^^,^^41^ Among the five studies, which reported on the baseline dysplastic status,^15^^,^^30^^,^^31^^,^^41^ only one study included patients with pure NDBE.^30^ The outcome of interest was EC in two studies,^12^^,^^34^ EAC in eight studies,^13^^,^^14^^,^^33^^,^^35–37^^,^^39^^,^^40^ and HGD/EAC in the remaining seven studies.^11^^,^^15^^,^^30–32^^,^^38^^,^^41^ Medication exposure was ascertained through record linkage with the pharmacy prescription database in nine studies.^12^^,^^13^^,^^32^^,^^34–37^^,^^39^^,^^40^ Three studies relied on medical record review.^30^^,^^38^^,^^41^ Two studies relied on self-reported use of medications.^14^^,^^33^ In the remaining two studies, the initial self-reported use of medications was cross-checked with the prescription record^15^^,^^31^ In the AspECT trial, patients were randomized in a 2 × 2 factorial design to receive either high-dose or low-dose PPI, with or without aspirin. Among eight studies, which defined BE based on endoscopic appearance of CLE, and presence of intestinal metaplasia, four studies required at least 2 cm of CLE^14^^,^^15^^,^^31^^,^^33^ and two studies required at least 1 cm^11^^,^^41^ to meet the criteria for BE definition. Only two studies did not report on the length of BE.^30^^,^^38^ Among 13 cohort and nested case–control studies, seven studies (54%) defined incident cases as cancers diagnosed after 12 months since the index diagnosis of BE.^12^^,^^13^^,^^30^^,^^32–34^^,^^38^ Two used a 9 month lag period for incident diagnosis of cancer,^15^^,^^31^ two used 6 months^37^^,^^41^ and two used 3 months.^35^^,^^36^

**Table 1:** Study characteristics

Study	Design	Location	Setting	CCM evaluated	NDBE at baseline (%)	Recruitment period	Definition of BE	No. of patients with NDBE/LGD at baseline	No of patients with HGD/EAC	Ascertainment of Drug Exposure	Outcome Definition	Lag period applied for incident case definition^*^	Median follow-up (years)	Variables adjusted for^‡^
Kastelein *et al*.^15^	PC	Netherlands	MC	PPI	NDBE-464 (86%) LGD-76 (14%)	2003–2004	IM,CLE ≥ 2 cm	540	40	Questionnaire+hospital records	HGD/EAC	9 months	5.2	1–7, 9–11
Kastelein *et al.*^31^	PC	Netherlands	MC	Aspirin, Statin	NDBE-491 (86%) LGD-79 (14%)	2003–2005	IM,CLE ≥ 2 cm	570	38	Questionnaire+hospital records	HGD/EAC	9 months	4.5	1–8, 9
Krishnamoorthi *et al.*^12^	RC	United Kingdom	PB	PPI, Statin	NR	1991–2010	READ code	9660	103	Primary care Database	OC	12 months	4.8	1–3,5–8, 12–15
Hvid-Jensen *et al.*^32^	nCC	Denmark	PB	PPI	NR	1995–2009	SNOMED Code+ IM	1437	140	Danish prescription database	HGD/EAC	12 months	5.7	1,2,4,5–7, 16
Masclee *et al.*^13^	nCC	UK	PB	PPI, Aspirin, Statin	NR	1996–2011	READ Code	777	45	Primary care database	EAC	12 months	4.2^†^	1–3,10,11, 17,18
Masclee *et al*.^13^	nCC	Netherlands	PB	PPI, Aspirin, Statin	NR	1996–2012	ICPC Code	1466	57	Primary care database	HGD/EAC	12 months	3.8^†^	1–3,10,11, 17,18
Tan *et al*.^36^	nCC	US	PB	PPI	NR	2004–2009	ICD-9 Code	1098	300	Veterans affairs datasets	EAC	3 months	NR	1–3,5–8, 12,16,19
Loomans-Kropp *et al*.^40^	nCC	US	PB	PPI, Statin	NR	2007–2013	ICD-9 Code	894	394	SEER-Medicare	EAC	NR	NR	1,2
Kambhampati *et al*.^41^	RC	US	SC	Statin	NDBE-441 (96%), LGD 19 (4%)	1992–2013	IM, CLE ≥ 1 cm	460	133	Hospital records	HGD/EAC	6 months	7.78^†^	1,3,4,9, 12,20–22
Thota *et al*.^38^	RC	US	SC	PPI	NDBE-843(82.2%) LGD-182 (17.8%)	2002–2015	IM, CLE (length not specified)	1025	57	Hospital records	HGD/EAC	12 months	3.67	1,2,9,13
Nguyen *et al*.^35^	nCC	US	PB	Statin	NR	2004–2011	ICD-9 Code	1167	311	Veterans affairs datasets	EAC	3 months	NR	1,2,3,5,7, 8,16
Cooper *et al*.^34^	nCC	UK	PB	Aspirin, Statin	NR	1988–2006	READ-code	3749	55	Primary care database	OC	12 months	4	1–3
Gatenby *et al*.^30^	RC	UK	MC	Aspirin	NDBE-736 (100%)	NR	CLE	736	30	Hospital records	HGD/EAC	12 months	NR	1,2,11
Nguyen *et al*.^37^	nCC	US	PB	PPI	NR	2000–2004	ICD-9 Code	812	116	Veterans affairs datasets	EAC	6 months	NR	1,2,5–8, 19
Jankowski *et al*.^11^	Randomized Trial	UK, Canada	MC	PPI, aspirin	NR	2005–2009	CLE ≥ 1 cm	2280	70	NA—allocated intention to treat	HGD/EAC	NA	8.9	NA
Beales *et al*. ^33^	CC	UK	SC	Aspirin, Statin	NR	2009–2011	IM, CLE ≥ 2 cm	255	85	Interview	EAC	12 months	NR	1–3,5–7, 12,18
De Jonge *et al*.^14^	CC	Netherlands	MC	PPI	NR	2003–2005	IM, CLE ≥ 2 cm	335	91	Questionnaire	EAC	NR	NR	1–3,5,6, 18
Agrawal *et al*.^39^	CC	US	PB	PPI, Statin	NR	1992–2012	ICD-9 Code	583	115	Veterans affairs datasets	EAC	NR	NR	1–3,5,7,8, 12,13–15

CC, case control study; CLE, columnar-lined esophagus; HGD, high-grade dysplasia; IM, intestinal metaplasia; MC, multicenter; EAC, esophageal adenocarcinoma; nCC; nested Case Control study; NR, not reported; NDBE, non-dysplastic Barrett’s esophagus; RC, retrospective cohort; SC, single center; UK, United Kingdom.

^*^Minimum time interval between diagnosis of BE and HGD/EAC/EC.

^†^Mean reported when median not available.

^‡^1. Age; 2. gender; 3. smoking; 4. Baseline LGD; 5. NSAID; 6. aspirin; 7. statin; 8. PPI; 9. length of BE; 10. esophagitis; 11. Year of BE diagnosis; 12. BMI; 13. hiatus hernia; 14. DM medications; 15. Type 2 Diabetes Mellitus; 16. H_2_ blockers; 17. gastritis; 18. alcohol history; 19. Ethnicity; 20. Family history of cancer; 21. Regurgitation; 22. Anemia.

### Participant characteristics

Participant characteristics are shown in supplementary Table S3. Of all participants, 74.7% were male. The mean age in the studies range from 58 to 71.2 years. Among the 14 studies that reported on the use of NSAIDs,^12–15^^,^^31–38^^,^^40^^,^^41^ 49.5% of participants were NSAID users. Mean BMI was reported in six studies.^11^^,^^13^^,^^14^^,^^33^^,^^34^^,^^38^ Mean BMI ranged from 25.8 to 28.9. Smoking status was recorded in 12 studies, where 46% of participants were smokers (current/ex-smokers).^11–15^^,^^31^^,^^33–36^^,^^39^^,^^41^ Four studies provided information on the number of patients diagnosed with GERD prior to BE diagnosis, where 86% (95% CI 85%–87%) had a prior diagnosis of GERD.^15^^,^^35^^,^^36^^,^^41^

### Association between PPI use and risk of malignant progression of BE

**Fig. 2:**
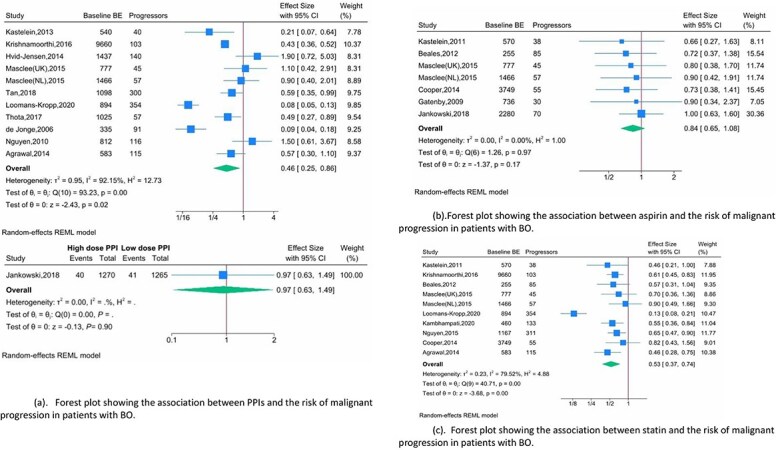
Meta-analysis assessing the association between PPIs, aspirin, and statin and the risk of malignant progression in patients with BE. (a). Forest plot showing the association between PPIs and the risk of malignant progression in patients with BE. (b) Forest plot showing the association between aspirin and the risk of malignant progression in patients with BE. (c) Forest plot showing the association between statin and the risk of malignant progression in patients with BE.

Eleven cohort studies (including nested case–control studies) reported on the effect of PPI (PPI use vs. no use) on the malignant progression in patients with BE.^12–15^^,^^32^^,^^36–40^ Reported effect sizes ranged from an adjusted OR of 0.09 (95% CI 0.05–0.2) to 1.9 (95% CI 0.7–4.9).^14^^,^^32^ The pooled results from the meta-analysis demonstrated that use of PPI was significantly associated with a 54% lower odds of malignant progression of BE (adjusted OR 0.46; 95% CI 0.25–0.86; *P* = 0.02; *I^2^* = 92.15%; *n* = 18,627; very low certainty) (Fig. 2a). We did not include the results from the AspECT trial in this meta-analysis as it compared the effect of high-dose PPI (esomeprazole 40 mg twice-daily) vs. low-dose PPI (esomeprazole 20 mg once-daily). The results from the AspECT trial and the pooled results from the meta-analysis of the observational studies are shown in Fig. 2a.

### Association between aspirin use and risk of malignant progression of BE

The effect of aspirin on the malignant progression of BE was reported in six studies.^11^^,^^13^^,^^30^^,^^31^^,^^33^^,^^34^ Reported effect sizes varied from an adjusted OR of 0.66 (95% CI 0.27–1.65) to 1.00 (95% CI 0.62–1.58).^11^^,^^31^ The meta-analysis demonstrated that aspirin demonstrated no significant association with malignant progression (adjusted OR 0.84; 95% CI 0.65–1.08; *P* = 0.17; *I^2^* = 0; *P* = 0.17; *n* = 9833; very low certainty) (Fig. 2b).

### Association between statin use and risk of malignant progression of BE

The effect of statins on malignant progression of BE was reported in nine studies.^12^^,^^13^^,^^31^^,^^33–35^^,^^39–41^ Reported effect sizes varied from an adjusted OR of 0.13 (95% CI 0.08–0.21) to 0.9 (95% CI 0.5–1.7).^13^^,^^40^ The meta-analysis demonstrated that statin use was associated with a 47% reduction in the odds of malignant progression of BE (adjusted OR 0.53; 95% CI 0.37–0.74; *P* < 0.001; *I^2^* = 79.52; *n* = 19,581; very low certainty) (Fig. 2c).

### Subgroup analyses

We performed subgroup analyses based on study design, study setting, baseline dysplasia status, study outcome, method of drug exposure assessment, definition of BE, length of BE, and overall RoB assessment (Table 2, Supplementary Figs S4–S27). Significant heterogeneity in the association between PPI use and risk of malignant progression was partly explained by the method of exposure assessment (database vs. review of medical records vs. self-report; OR 0.62; 95% CI 0.31–1.24; number of studies = 8; *I^2^* = 92.24% vs OR 0.37; 95% CI 0.17–0.81; number of studies = 2; *I*^2^ = 41.47% vs OR 0.09; 95% CI 0.04–0.18; number of studies = 1; *P*_interaction_ < 0.001) (Supplementary Fig. S8). Baseline dysplastic status (NDBE vs. at most LGD vs. not reported) had no effect on the effect sizes for all three medications (Supplementary Figs S6, S14, S22). There were no significant differences between studies, where BE was defined based on the presence of CLE of at least 1 cm vs. length not reported (Supplementary Figs S11, S19, S27). The association remained consistent across all other subgroups. Inverse associations with all three medications for malignant progression were numerically stronger in studies at critical RoB compared to those at serious RoB in subgroup analyses, although did not reach statistical significance (Table 2, Supplementary Figs S9, S16, S25).

**Table 2:** Subgroup analyses

Groups	Categories	No of studies	Odds ratio (95% CI)	Heterogeneity within groups (*I*^2^)	Heterogeneity between groups (*P interaction*)
PPI					
Study design	Case–control	2	0.23 (0.04–1.39)	93.06%	0.37
	Cohort	9	0.55 (0.28–1.05)	91.52%	
Study Setting	Hospital-based	3	0.21 (0.08–0.60)	81.52%	0.10
	Population-based	8	0.62 (0.31–1.24)	92.24%	
Baseline dysplasia	Not reported	9	0.50 (0.24–1.05)	93.79%	0.58
	At most LGD	2	0.37 (0.17–0.81)	41.47%	
Study outcome	HGD/EAC	4	0.66 (0.29–1.51)	74.06%	0.60
	EC	1	0.43 (0.36–0.52)	––	
	EAC	6	0.39 (0.14–1.05)	92.84%	
Risk of bias assessment	Critical	3	0.30 (0.09–0.93)	89.46%	0.37
	Serious	8	0.56 (0.26–1.17)	92.47%	
Exposure ascertainment	Medical records	2	0.37 (0.17–0.81)	41.47%	<0.0001
	Self-report	1	0.09 (0.04–0.18)	52.19%	
	Database	8	0.62 (0.31–1.24)	92.24%	
Definition of BE	Biospy-confirmed	3	0.21 (0.08–0.60)	81.52%	0.10
	Coded diagnosis of BE	8	0.62 (0.31–1.24)	92.24%	
BE length	At least 1 cm	3	0.22 (0.07–0.68)	83.09%	0.14
	Not reported	8	0.60 (0.30–1.22)	92.44%	
Aspirin					
Study design	Case–control	1	0.72 (0.37–1.38)	––	0.65
	Cohort	5	0.79 (0.56–1.12)	0.00%	
	RCT	1	1.00 (0.63–1.60)	––	
Study setting	Hospital Based	4	0.86 (0.62–1.20)	0%	0.79
	Population based	3	0.80 (0.53–1.21)	0%	
Baseline dysplasia	NDBE	1	0.90 (0.34–2.37)	––	0.86
	Not reported	5	0.85 (0.64–1.12)	0%	
	At most LGD	1	0.66 (0.27–1.63)	––	
Study outcome	HGD/EAC	4	0.91 (0.65–1.28)	0%	0.75
	EAC	2	0.75 (0.46–1.23)	0%	
	EC	1	0.73 (0.38–1.41)	––	
Exposure ascertainment	Database	3	0.80 (0.53–1.21)	0%	0.96
	Medical record	2	0.76 (0.39–1.48)	0%	
	Self-report	1	0.72 (0.37–1.38)	––	
Risk of bias assessment	Critical	3	0.74 (0.46–1.18)	0%	0.81
	serious	3	0.80 (0.53–1.21)	0%	
Definition of BE	Biospy-confirmed	3	0.85 (0.60–1.21)	0%	0.96
	Coded diagnosis of BE	3	0.80 (0.53–1.21)	0%	
	Endoscopic appearance only	1	0.90 (0.34–2.37)	––	
BE length	At least 1 cm	3	0.85 (0.60–1.21)	0%	0.85
	Not reported	4	0.81 (0.56–1.19)	0%	
Statins					
Study design	Case–control	2	0.50 (0.34–0.73)	0%	0.84
	Cohort	8	0.53 (0.35–0.82)	84.51%	
Study setting	Hospital-based	3	0.54 (0.39–0.74)	0%	0.94
	Population-based	7	0.53 (0.32–0.86)	87.03%	
Baseline dysplasia status	Not reported	8	0.53 (0.35–0.81)	84.28%	0.99
	At most LGD	2	0.53 (0.37–0.76)	0%	
Study outcome	HGD/EAC	3	0.61 (0.44–0.85)	8.05%	0.51
	EAC	5	0.43 (0.23–0.80)	87.18%	
	EC	2	0.64 (0.49–0.85)	0%	
Exposure ascertainment	Database	7	0.53 (0.32–0.86)	87.03%	0.98
	Medical record	2	0.53 (0.37–0.76)	0%	
	Self-report	1	0.57 (0.31–1.04)	––	
Length of BE	At least 1 cm	3	0.54 (0.39–0.74)	0%	0.94
	Not reported	7	0.53 (0.32–0.86)	87.03%	
Risk of bias assessment	Critical	4	0.35 (0.17–0.69)	82.76%	0.08
	Serious	6	0.65 (0.55–0.77)	0%	
Definition of BE	Biopsy-confirmed	3	0.54 (0.39–0.74)	0%	0.94
	Coded diagnosis of BE	7	0.53 (0.32–0.86)	87.03%	

EAC, esophageal adenocarcinoma; OC, esophageal cancer; HGD, high-grade dysplasia; LGD, low-grade dysplasia; RCT, randomized controlled-trial.

### Risk of bias

The assessment of domain-specific as well as overall RoB in each included study is shown in Supplementary Tables S4,S5. The assessment of overall RoB using the ROBINS-I tool demonstrated that six studies were at critical RoB,^14^^,^^30^^,^^31^^,^^33^^,^^38^^,^^39^ while the remaining 10 studies were at serious RoB (Supplementary Table S4).^12^^,^^13^^,^^15^^,^^32^^,^^34–37^^,^^40^^,^^41^ Among the six studies, which were at a critical RoB, five were due to bias in selection of participants^14^^,^^30^^,^^31^^,^^33^^,^^39^ and one was due to confounding.^38^

**Fig. 3:**
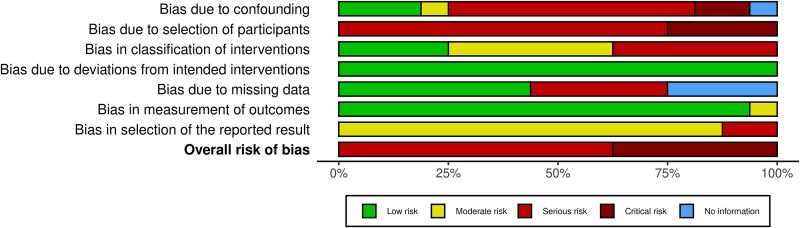
Weighted bar plots of the distribution of risk-of-bias judgments within each bias domain of the ROBINS-I tool using the ROBVIS (visualization tool).

All included studies were either at serious (11 studies) or critical (five studies) RoB due to selection of participants (Fig. 3). This was mainly contributed by prevalent-user and immortal-time bias. With regards to confounding, ten studies were at serious RoB,^12^^,^^13^^,^^15^^,^^31–34^^,^^37^^,^^40^^,^^41^ two were at critical risk,^30^^,^^38^ two at moderate risk,^14^^,^^39^ and two at low risk.^35^^,^^36^ All the included studies were considered to be at low RoB due to deviations from intended interventions as the causal effect of interest related to allocation to treatment at baseline and not post-baseline deviations (Fig. 3). The single trial included in our meta-analysis was considered to have an overall low RoB using the ROB 2 tool (Supplementary Table S5).^11^ The overall body of evidence was rated to be very low using the GRADE framework (Supplementary Table S6).

### Main sources of bias

Thirteen studies were considered at serious or critical RoB due to confounding at baseline and/or time-varying confounding.^12^^,^^14^^,^^15^^,^^30–34^^,^^36–38^^,^^40^^,^^41^ All the included studies were considered to be affected by prevalent user bias due to inclusion of prevalent users of aspirin, statin, and PPI. Five studies were considered to be affected by immortal-time bias.^30^^,^^31^^,^^34^^,^^38^^,^^41^ Among three case–control studies, two were at risk of time-window bias.^14^^,^^39^

### Publication bias

There were 11 studies included in the meta-analysis demonstrating the association between PPI use and risk of malignant progression of BE. There was no evidence of small-study effects, such as publication bias on visual inspection of funnel plot or Egger’s test (*P* = 0.27) (Supplementary Fig. S28).

## DISCUSSION

### Summary of findings

In summary, the observational research summarized in our systematic review demonstrated that PPIs and statins were significantly inversely associated with malignant progression of BE with impressive effect sizes (54% and 47% reduced odds, respectively), while aspirin use was not significantly associated. Additionally, all the observational studies included in our study were either at serious or critical RoB, with more extreme effect sizes observed in those studies at critical RoB compared to those at serious risk. The predominant sources of bias were confounding, prevalent user bias, immortal time bias, and relevant to case–control studies, time-window bias. Trial evidence demonstrated no significant difference between aspirin and PPI groups for the outcome of HGD/EAC. This study was at low RoB. Collectively, the overall GRADE certainty of evidence related to the role CCMs in BE was very low.

Prevalent user bias is a type of selection bias that arises when prevalent users (drug prescriptions or use initiated before baseline), instead of new (incident) users of a particular medication are included in the analyses. This results in an over-estimation of benefits and an apparent survival advantage in the prevalent user group because prevalent users, by definition, have survived under treatment, and individuals who experienced the outcome during the initial exposure period would not have survived to be included in the study.^42^^,^^43^ Immortal-time bias occurs when start of study follow-up, eligibility, and treatment initiation do not occur in alignment, and there is a delay between start of study follow-up and treatment initiation. Immortal time bias can arise when this ‘unexposed’ period is misclassified as ‘exposed’, resulting in a period of follow-up (‘immortal-time’) during which study outcomes or death cannot occur by definition, leading to an apparent but spurious protective effect of the intervention.^42^^,^^44^ Time-window bias is a methodological pitfall specific to case–control studies, which arises as a failure to match the time-windows between cases and controls used to define time-dependent exposures.^45^ This results in different durations of exposure periods between cases and controls, leading to biased estimates.

The meta-estimates from our study are compatible with the findings from previous meta-analyses on the effect of PPI, statins, and aspirin on the malignant progression of BE. The most up-to-date meta-analysis conducted by Yao *et al*. reported that use of PPI is associated with reduction in malignant progression of BE by 54% (adjusted RR 0.46; 95% CI 0.32–0.71; *P* < 0.001; *I^2^* = 78%).^46^ We did not include the two studies included in the previous meta-analyses as they were only available as conference abstracts, which limited our detailed assessment of RoB.^47^^,^^48^ Similar effect sizes were also reported in two further meta-analyses.^19^^,^^49^^,^^50^ Similarly, our findings corroborated the results from the meta-analysis by Thomas *et al.,* which demonstrated statin use was inversely associated with risk of malignant progression by 41% (adjusted OR 0.59; 95% CI 0.50–0.61; *P* < 0.0001; *I^2^* = 0%).^20^ This inverse association was also demonstrated in two further meta-analyses.^51^^,^^52^ Likewise, the results of a meta-analysis by Thrift *et al*. showed no association between aspirin use and malignant progression of BE.^53^ A meta-analysis by Zhang *et al*., in contrast to ours, demonstrated significant inverse associations with aspirin use (adjusted OR 0.63; 95% CI 0.43–0.94).^21^ This difference is explained by the inclusion in our review of additional studies, including trial evidence. All previous systematic reviews used the NOS for quality assessment, which showed that 35% of the included studies were of moderate quality and 64% were of high quality.^19^^,^^20^^,^^49–51^ Despite its ease-of-use, it does not cover important bias domains, which are highly relevant to NRSI.^54^ It is also susceptible to inter-observer variability due to a lack of comprehensive manual with clear instructions.^55^

The AspECT trial is a landmark study with a 2×2 factorial design including 2557 patients with BE.^11^ In the main intention-to-treat analysis, this trial demonstrated that high-dose PPI in combination with aspirin was superior to low-dose PPI with no aspirin for preventing the composite end point of all-cause mortality, HGD, or EAC (TR = 1.59; 95% CI 1.14–2.23; *P* = 0.0068). While the AspECT trial provides the most robust data to date regarding the role of aspirin and PPIs in reducing the rate of adverse outcomes in patients with BE, the benefits seemed to be driven by a reduction in all-cause mortality, rather than EAC, the major endpoint of Barrett’s chemoprevention (association between high-dose PPI vs. low-dose PPI and development of EAC; HR 0.97; 95% CI 0.63–1.49; *P* = 0.90; association between aspirin vs. no aspirin and development of EAC; HR 1.00; 95% CI 0.63–1.59; *P* = 0.99). Assuming aspirin and PPIs exert chemopreventive effects in patients with BE, there are several potential reasons the AspECT trial did not demonstrate clinically apparent benefit with the use of these drugs. More than half (52%) of participants initially allocated to the low-dose PPI (20 mg once daily) required higher doses of PPI (40 mg once daily) at 8 years, resulting in substantial contamination of the exposure in the context of the primary intention-to-treat analysis and therefore reduced gradient between the dose exposures. Similarly, only 57% of the initial participants allocated to aspirin remained on aspirin at 8 years. Additionally, comparisons were not powered for the outcome of EAC alone (the major outcome for Barrett’s chemoprevention). Per-protocol analysis (with populations defined based on 6 and 12 month’s use of aspirin and esomeprazole, respectively), demonstrated no significant differences between groups. However, these analyses were very likely under powered and did not account for post-randomization confounding and selection bias, and therefore drawing firm inferences on sustained use strategies from this trial is not possible.^56^

Considering the very low certainty of the overall evidence base (from observational and interventional research) for the clinical efficacy of CCMs, there is little direct clinical justification for recommending these drugs for Barrett’s chemoprevention. In line with this, the recently reported NICE guidelines did not recommend the use of PPIs or aspirin as chemoprevention in BE.^18^ NICE acknowledge that although treatment with PPIs might confer chemopreventive benefits compared to no PPI, demonstrating this in a trial setting would be challenging as most patients with BE need treatment for control of reflux symptoms.^18^ In contrast, the ESGE guidelines advocated the use of PPIs for chemoprevention, a weak recommendation based on moderate quality evidence.^16^ The ACG also recommended at least once daily PPI as chemoprevention given the unclear benefit of higher doses of PPI on oncogenesis.^17^

### Strengths and limitations of our study

Our systematic review has a number of strengths. The study protocol was pre-registered. Our study provides the most contemporaneous review on the clinical evidence reporting the assumed causal association between aspirin, PPI, and statins and malignant progression of BE. To the best of our knowledge, our systematic review is the first to provide a comprehensive assessment of RoB of the studies reporting on the effect of CCMs on malignant progression of BE using the ROBINS-I and RoB 2 tools. The ROBINS-I tool incorporates the concept of causal inference based on counterfactual reasoning and provides a structured approach to evaluating the RoB in the results of NRSI by considering each NRSI as an attempt to emulate a hypothetical trial.^22^ It is the only tool advocated by the Cochrane Handbook for evaluating bias for non-randomized studies.^57^ The certainty of evidence was evaluated using the GRADE Approach which offers a structured method for rating the quality of evidence and making clinical practice recommendations.^26^

Our systematic review has some limitations. First, while our study focuses on the full-text published literature on the role of chemoprevention in BE, it did not capture all potentially relevant evidence, for example, the grey literature. However, this study was intended to enable a detailed assessment of RoB which is predicated on the availability of study detail only provided in full-text publications rather than abstracts. Reassuringly, our analysis indicated a low risk of small study effects including publication bias. Second, our review did not focus on sustained use comparisons, which is of direct clinical relevance in this context. Additionally, while the ROBINS-I tool is a contemporaneous and rigorous tool which aims to assess RoB based on counterfactual reasoning, it has been criticized for its conceptual complexity and challenges associated with application of its domains consistently across different studies. We therefore acknowledge that no RoB tool is perfect.^58^

Third, a key limitation of our study is the inability to include only patients with confirmed NDBE, and inclusion of cohorts with uncertain dysplastic status. Only one study exclusively included patients with NDBE at baseline, and four included mixed cohorts of NDBE and LGD (overall 86% had NDBE). The results are therefore most applicable to cohorts predominantly with NDBE at baseline. Furthermore, the eight remaining population-based studies defined BE using diagnostic codes without detailed histologic information on baseline dysplasia status. However, it is expected the majority of patients in these studies had NDBE at baseline.^59^ Importantly, including these studies offered key strengths such as nationally representative cohorts, well-defined drug exposures from prescription data, and unbiased outcome ascertainment.

In addition, patients with confirmed LGD at baseline are expected to be at higher risk of both prevalent and incident neoplasia,^60^ which may introduce confounding if baseline dysplasia status is differentially distributed by CCM use. Reassuringly, there was no evidence to suggest this from two studies which reported baseline LGD status stratified by PPI (vs. no PPI) and statin (vs. no statin) use.^15^^,^^31^ Furthermore, three of the four studies including known baseline LGD adjusted for baseline LGD status.^15^^,^^31^^,^^41^ Finally, among the eight studies which defined BE based on endoscopic appearance of CLE, two did not specify the length of BE. However, subgroup analyses investigating the effect of length of BE on the overall effect sizes did not demonstrate any significant differences.

### Implications and recommendations

Whether these CCMs materially reduce the incidence of EAC in patients with BE remains unclear. While previous observational studies suggest that use of CCMs might prevent cancer, the protective effects are likely over-estimates due to serious or critical RoB. The methodology employed in the included studies was often state-of-the-art at the time of publication, and we acknowledge that our RoB assessments were based on contemporaneous guidance and practice which was not available at that time. According to clinicaltrials.org, there are no ongoing trials of Barrett’s chemoprevention. Conducting future trials on Barrett’s chemoprevention would be challenging due to a variety of reasons, including a low event rate (given low overall rates of progression to cancer), prevalent use of these CCMs leading to difficulties in comparing the overall net benefit of using CCMs with no use, and issues with drug adherence leading to contamination of treatment groups. PPI treatment, in particular, is driven by symptom severity and closely correlated with outcomes, resulting in intractable confounding. Given that symptomatic PPI use is almost universal in patients with BE, the value of further evaluation in this context may be limited.

The application of the ROBINS-I tool provides a valuable framework for evaluating the validity of observational studies investigating Barrett’s chemoprevention. This is of direct clinical relevance, as it is essential to understand the validity of the evidence that underpins guideline recommendations, particularly given the discrepancies between current guideline recommendations. In this context, we recommend the conduct of future studies to evaluate the causal effect of CCMs on the malignant progression of BE that leverage large representative healthcare databases (with robust capture of exposures, covariates and outcomes), and advancements in comparative effectiveness methodology for causal estimation of treatment effects.^61^ Sustained drug use comparisons which account for time-dependent confounding and selection bias would likely be of particular clinical interest given assumed prolonged induction period for Barrett’s carcinogenesis.^62^

Use of alternative strategies, such as anti-reflux surgery, has been explored to prevent malignant progression in patients with BE. A multinational population-based cohort study including 33,939 patients with BE found that patients who received anti-reflux surgery did not have reduced risk of malignant progression compared to use of medications alone. Instead, the risk was found to increase throughout the follow-up among patients who underwent surgery.^63^ This finding corroborated with the findings from previous systematic reviews on the malignant progression of BE, which did not find any significant reduction in risk of EAC in patients who received anti-reflux surgery.^64^ In line with this, various clinical guidelines recommend against the use of anti-reflux surgery as an anti-neoplastic measure in BE.^17^^,^^18^^,^^65^

## CONCLUSION

In conclusion, published observational research demonstrates statins and PPIs are significantly inversely associated with HGD/EAC in patients with BE, while aspirin use is not. All observational studies were either at serious or critical RoB. Trial evidence was at low RoB and did not demonstrate any significant differences between groups for aspirin and PPI comparisons for the outcome of HGD/EAC. Despite biological plausibility, given the very low certainty of evidence, there is little rationale to recommend chemoprevention with PPIs, aspirin, or statins in patients with BE.

## Supplementary Material

Manuscript_Submission_Supplementary_DisE_Revised_01_07_doaf062

## Data Availability

The dataset is available from the corresponding author.

## Notes

Specific author contributions: Mie Thu Ko (Data curation, Formal analysis, Software, Writing—original draft), Agha Rizwanullah (Data curation, Writing—review & editing), Zain Jafri (Data curation, Writing—review & editing), Adriel Fung (Data curation, Writing—review & editing), Leo Alexandre (Conceptualization, Data curation, Formal analysis, Project administration, Supervision, Writing—review & editing).

Financial support: Mie Thu Ko, NIHR Academic Clinical Fellow, is funded by the National Institute for Health Research (NIHR) for this research project. The views expressed in this publication are those of the authors and not necessarily those of the NIHR, NHS, or the UK Department of Health and Social Care.
