# Supplementary material for: Chemoprevention of Barrett’s Esophagus: a Systematic Review and Comprehensive Assessment of Bias
Source: Dis Esophagus. 2025 Aug 6;38(4):doaf062. doi: 10.1093/dote/doaf062 (PMC12341868; doi:10.1093/dote/doaf062)
Supplement: Manuscript_Submission_Supplementary_DisE_Revised_01_07_doaf062 [file manuscript_submission_supplementary_dise_revised_01_07_doaf062.docx]

Supplementary Appendix

Table of Contents

[Supplementary Table 1. Search Strategy 3](#_Toc196493592)

[Supplementary Table 2. Studies Selected for Full Text Review which did not meet eligibility 4](#_Toc196493593)

[Supplementary Table 3. Participant characteristics 6](#_Toc196493594)

[Supplementary Table 4. Risk of Bias Assessment of the included observational studies using the ROBINS-I Tool 8](#_Toc196493595)

[Supplementary Table 5. Risk of Bias Assessment of the AsPECT Trial using the ROB-2 Tool. 9](#_Toc196493596)

[Supplementary Figure 1. “Traffic light” Plots of the domain-level judgements for each included observational study using the ROBVIS( visualization tool 10](#_Toc196493597)

[Supplementary Figure 2. “Traffic light” Plots of the domain-level judgements for the AspECT Trial using the ROBVIS( visualization tool) 11](#_Toc196493598)

[Supplementary Figure 3 . Weighted bar plots of the distribution of risk-of-bias judgements within each bias domain of the ROB2 tool using the ROBVIS ( visualization tool) 11](#_Toc196493599)

[Supplementary Table 6. GRADE summary of findings table. Use of statins, aspirin and proton pump inhibitors compared with no use in patients with Barrett’s esophagus for the outcome of high-grade dysplasia or cancer. 12](#_Toc196493600)

[Supplementary Figure 4. Subgroup Analysis Based on Study Design (PPI) 14](#_Toc196493601)

[Supplementary Figure 5. Subgroup Analysis Based on Study Setting (PPI) 15](#_Toc196493602)

[Supplementary Figure 6 . Subgroup Analysis Based on baseline dysplastic status (PPI) 16](#_Toc196493603)

[Supplementary Figure 7. Subgroup Analysis Based on Study Outcome(PPI) 17](#_Toc196493604)

[Supplementary Figure 8. Subgroup Analysis Based on exposure ascertainment(PPI) 18](#_Toc196493605)

[Supplementary Figure 9. Subgroup Analysis Based on risk of bias assessment(PPI) 19](#_Toc196493606)

[Supplementary Figure 10. Subgroup Analysis Based on definition of BE(PPI) 20](#_Toc196493607)

[20](#_Toc196493608)

[Supplementary Figure 24. Subgroup Analysis Based on BE length (PPI) 21](#_Toc196493609)

[Supplementary Figure 12. Subgroup Analysis Based on Study Design(aspirin) 22](#_Toc196493611)

[Supplementary Figure 13. Subgroup Analysis Based on study setting ( aspirin) 23](#_Toc196493612)

[Supplementary Figure 14. Subgroup Analysis Based on Baseline Dysplastic Status(aspirin) 24](#_Toc196493613)

[Supplementary Figure 15. Subgroup Analysis Based on study outcome(aspirin) 25](#_Toc196493614)

[Supplementary Figure 16. Subgroup Analysis Based on exposure ascertainment (aspirin) 26](#_Toc196493615)

[Supplementary Figure 17. Subgroup Analysis Based on risk of bias assessment(aspirin) 27](#_Toc196493616)

[Supplementary Figure 18. Subgroup Analysis Based on BE definition (aspirin) 28](#_Toc196493617)

[Supplementary Figure 19. Subgroup Analysis Based on BE length (aspirin) 29](#_Toc196493618)

[29](#_Toc196493619)

[Supplementary Figure 20. Subgroup Analysis Based on study design(statin) 30](#_Toc196493620)

[Supplementary Figure 21. Subgroup Analysis Based on Study Setting ( statin) 31](#_Toc196493621)

[Supplementary Figure 22 . Subgroup Analysis Based on Baseline Dysplastic Status( statin) 32](#_Toc196493622)

[Supplementary Figure 23. Subgroup Analysis Based on study outcome(Statin) 33](#_Toc196493623)

[Supplementary Figure 24. Subgroup Analysis Based on exposure ascertainment(statin) 34](#_Toc196493624)

[Supplementary Figure 25. Subgroup Analysis Based on risk of bias assessment(statin) 35](#_Toc196493625)

[Supplementary Figure 26. Subgroup Analysis Based on BE definition(statin) 36](#_Toc196493626)

[Supplementary Figure 27. Subgroup Analysis Based on BE length (statin) 37](#_Toc196493627)

[Supplementary figure 28. Funnel plot to assess publication bias 38](#_Toc196493628)

[Supplementary figure 29: Directed Acyclic Graph (DAG): the assumed causal relationships between PPIs and malignant progression of Barrett’s esophagus. 39](#_Toc196493629)

[Supplementary figure 30: Directed Acyclic Graph (DAG): the assumed causal relationships between aspirin and malignant progression of Barrett’s esophagus. 41](#_Toc196493630)

[Supplementary figure 31: Directed Acyclic Graph (DAG): the assumed causal relationships between statins and malignant progression of Barrett’s esophagus. 42](#_Toc196493631)

# Supplementary Table 1. Search Strategy

1. ((barret$ or columnar) adj1 (esophag$ or oesophag$ or metaplasia)).ab,hw,kw,ti.
2. ((columnar adj1 lined) and (esophag$ or oesophag$)).ab,hw,kw,ti.
3. Barrett Esophagus/
4. (dysplasia or cancer or carcinoma or adenocarcinoma or neoplas$ or malignan$ or progression or progressor$).ab,hw,kw,ti.
5. (observational or epidemiologic$ or case-control or patients or cohort$ or cross-section$ or retrospective or prospective$ or trial).ab,hw,kw,ti.
6. (statin$ or simvastatin or atorvastatin or pravastatin or proton pump inhibitor$ or PPI$ or omeprazole or esomeprazole or lansoprazole or pantoprazole or aspirin or chemoprevent$).ab,hw,kw,ti.
7. or/1-3
8. and/4-7
9. (conference abstract or editorial or erratum or note or news).st,mp. or (case report or expert review or case series or consensus).ti.
10. 8 not 9
11. remove duplicates from 10

#

#

# Supplementary Table 2. Studies Selected for Full Text Review which did not meet eligibility

| **Study,year** | **Reason Excluded** |
| --- | --- |
| MEDLINE and Embase Search | |
| Beales,2016^1^ | Inclusion criteria 2 not met. Outcome was not HGD, EAC or esophageal cancer. |
| Brown,2015^2^ | Inclusion criteria 2 not met. Outcome was not HGD, EAC or esophageal cancer. |
| Beales,2013^3^ | Inclusion criteria 1 not met. HGD at baseline. |
| Kantor,2012^4^ | Inclusion criteria 1 not met. HGD at baseline. |
| Hillman,2008^5^ | Inclusion criteria 2 not met. Outcome was not HGD, EAC or esophageal cancer. |
| Vaughan,2005^6^ | Inclusion criteria 1 not met. HGD at baseline. |
| Hillman,2004^7^ | Inclusion criteria 2 not met. Outcome was not HGD, EAC or esophageal cancer. |
| El-Serag,2004^8^ | Inclusion criteria 2 not met. Outcome was not HGD, EAC or esophageal cancer. |
| Gatenby,2009^9^ | Inclusion criteria 3 not met. Comparisons did not include PPI vs no PPI. |
| Nguyen,2009^10^ | Inclusion criteria 1-3 met, but overlapping population with more contemporaneous cohort. |
| Cooper,2006^11^ | Inclusion criteria 2 not met. Outcome was not HGD, EAC or esophageal cancer |
| Tsibouris,2004^12^ | Inclusion criteria 3 not met. Drug exposures did not include aspirin, PPI, or statins. |
| Galipeau,2007^13^ | Inclusion criteria 2 not met. Outcome was not HGD, EAC or esophageal cancer |
| Ortiz,1996^14^ | Inclusion criteria 2 not met. Outcome was not HGD, EAC or esophageal cancer. |
| Babic,2005^15^ | Inclusion criteria 2 not met. Outcome was not HGD, EAC or esophageal cancer |

Eligibility criteria:

Inclusion criteria

1. Documented BE (either purely non-dysplastic or a mixed cohort of non-dysplastic BE and LGD or indefinite for neoplasia or unknown dysplasia status) at entry for cohort studies or as the control group (who did not progress to HGD/EAC/esophageal cancer (OC) [histological subtype not specified]) in case-control studies
2. Reported outcome of HGD, EAC or esophageal cancer
3. Drug exposures and comparisons include PPI, statin or aspirin use compared with no use, and higher-dose use compared with lower-dose use. We did not put any restrictions on the minimum length of columnar-lined esophagus or whether intestinal metaplasia was required for the definition of BE.

Exclusion criteria

1. Presence of HGD or EAC at baseline
2. Effect sizes or data necessary to calculate effect sizes not reported

# Supplementary Table 3. Participant characteristics

| Study | Age, mean (SD) | Sex(%men) | BMI(mean) | NSAID(%) | Smoking(%) | Patients diagnosed with GERD (%) |
| --- | --- | --- | --- | --- | --- | --- |
| Kastelein,2013 | 60.5(53.4-67.8) | 71 | NR | 6 | 19 | 27 |
| Kastelein,2011 | 60.4(IQR 14.5)^a^ | 72 | NR | 56 | 66 | 30 |
| Krishnamoorthi,2016 | 63(13.5) | 62.6 | NR | 62.27 | 51.9 | NR |
| Hvid-Jensen,2014 | 67.7(NR)^a^ | 67.3 | NR | 17.1 | NR | NR |
| Masclee(UK),2015 | 71.2(10.4) | 91 | 27.7(4.1) | 27.5 | 51 | NR |
| Masclee(NL),2015 | 68.8(8.2) | 70 | 28.9(6.8) | 11.8 | 49.5 | NR |
| Tan,2018 | 64.8(9.2) | 100 | NR | 40.3 |  | 71.7 |
| Loomans-Kropp,2020 | NR | 76.8 | NR | 12.7 | NR | NR |
| Kambhampati,2020 | 68.51(13.03) | 64 | NR | 13.7 | 49 | 98.26 |
| Thota,2017 | 59.0(12.6) | 71.9 | 27.9(7.7) | 57.3 | NR | NR |
| Nguyen,2015 | 64.7(9.1) | 100 | NR | 23.8 | 13.4 | 70.4 |
| Cooper,2014 | 63(IQR 52-72) | 63 | 25.8 (IQR 23.7–28.5) | 69 | 55 | NR |
| Gatenby,2009 | 64.5 | NR | NR | NR | NR | NR |
| Nguyen,2010 | 65.0(10.3) | 97.4 | NR | 49.1 | NR | NR |
| Jankowski,2018 | 59(PPI), 58(aspirin) | 80(PPI)  79(aspirin) | 27 (IQR 25-30) | NR | 50 | NR |
| Beales,2012 | 67.3 (12.0) | 80 | 27.8(7.2) | 3.5 | 59.5 | NR |
| De Jonge,2006 | 62 ( 11.7) | 67 | 26 (5.7) | 55 | 74 | NR |
| Agrawal,2014 | 61.4(11.4) | 100 | NR | NR |  | NR |

^a^ =median reported when mean not available

# Supplementary Table 4. Risk of Bias Assessment of the included observational studies using the ROBINS-I Tool

| Study | D1 | D2 | D3 | D4 | D5 | D6 | D7 | Overall |
| --- | --- | --- | --- | --- | --- | --- | --- | --- |
| Kastelein, 2013 | Serious | Serious | Moderate | Low | Serious | Low | Moderate | Serious |
| Kastelein, 2011 | Serious | Critical | Moderate | Low | Serious | Low | Moderate | Critical |
| Krishnamoorthi, 2016 | Serious | Serious | Low | Low | Serious | Low | Moderate | Serious |
| Hvid-Jensen, 2014 | Serious | Serious | Low | Low | Low | Low | Moderate | Serious |
| Beales, 2012 | Serious | Critical | Serious | Low | Serious | Moderate | Moderate | Critical |
| Masclee, 2015 | Serious | Serious | Low | Low | Low | Low | Moderate | Serious |
| Tan, 2018 | Low | Serious | Moderate | Low | Low | Low | Moderate | Serious |
| Loomans-Kropp, 2020 | Serious | Serious | Moderate | Low | Low | Low | Moderate | Serious |
| Kambhampati, 2020 | Serious | Serious | Serious | Low | No information | Low | Serious | Serious |
| Thota, 2017 | Critical | Serious | Serious | Low | Serious | Low | Serious | Critical |
| Nguyen, 2015 | Low | Serious | Moderate | Low | Low | Low | Moderate | Serious |
| Cooper, 2014 | Serious | Serious | Low | Low | No information | Low | Moderate | Serious |
| de Jonge, 2006 | Moderate | Critical | Serious | Low | Low | Low | Moderate | Critical |
| Gatenby, 2009 | Critical | Critical | Serious | Low | No information | Low | Moderate | Critical |
| Nguyen, 2010 | Serious | Serious | Moderate | Low | Low | Low | Moderate | Serious |
| Agrawal,2014 | Moderate | Critical | Serious | Low | No information | Low | Moderate | Critical |

Domains:

D1: Bias due to confounding

D2: Bias due to selection of participants

D3: Bias in classification of interventions

D4: Bias due to deviation from intended interventions

D5: Bias due to missing data

D6: Bias in measurement of outcomes

D7: Bias in selection of the reported result

# Supplementary Table 5. Risk of Bias Assessment of the AsPECT Trial using the ROB-2 Tool.

| Study | D1 | D2 | D3 | D4 | D5 | Overall |
| --- | --- | --- | --- | --- | --- | --- |
| Jankowski,2018 | Low | Low | Low | Low | Low | Low |

Domains:

D1: Bias arising from the randomization process

D2: Bias due to deviations from intended interventions

D3: Bias due to missing outcome data

D4: Bias in measurement of the outcome

D5: Bias in selection of the reported result

Supplementary Figure 1. “Traffic light” Plots of the domain-level judgements for each included observational study using the ROBVIS( visualization tool)


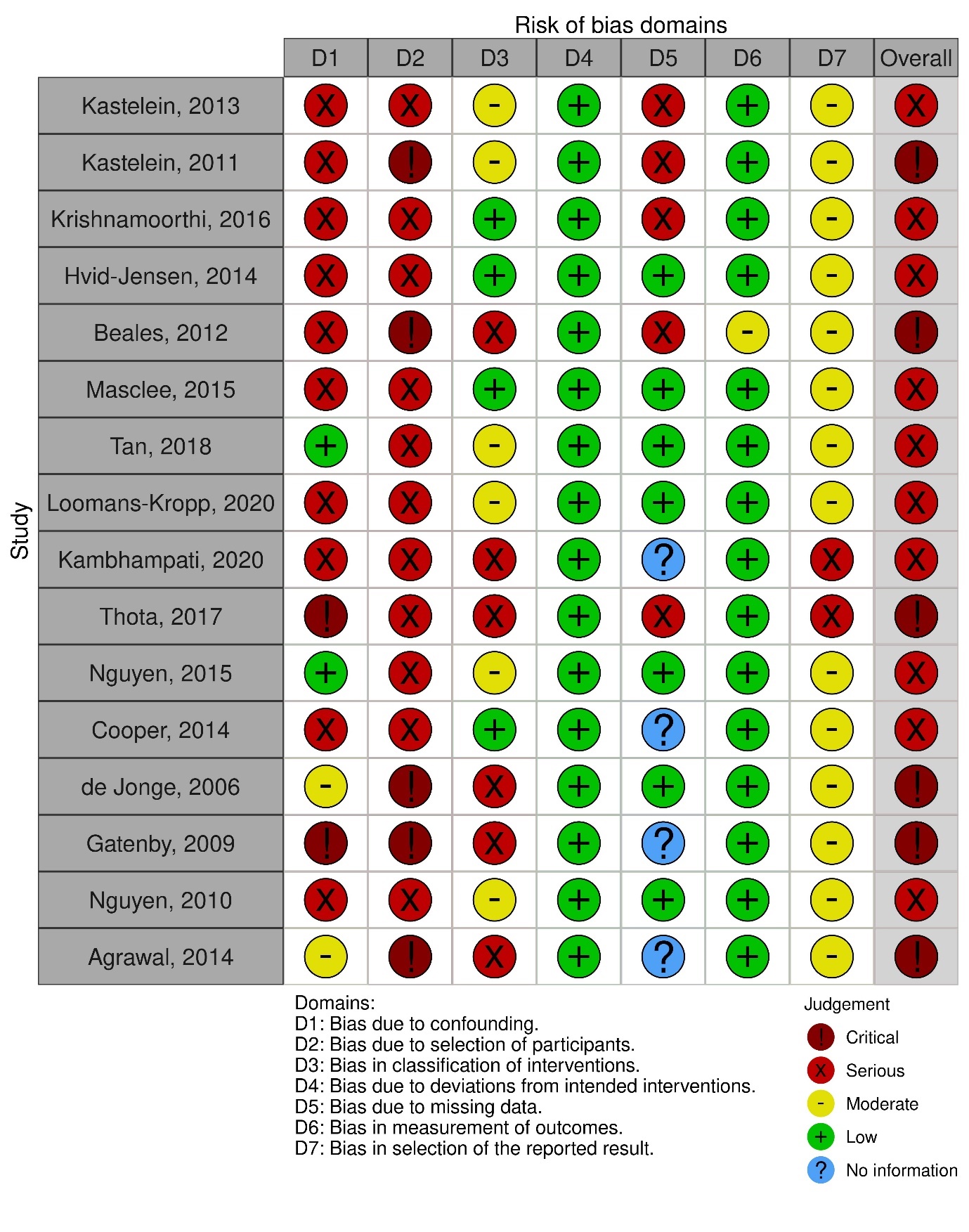


# Supplementary Figure 2. “Traffic light” Plots of the domain-level judgements for the AspECT Trial using the ROBVIS( visualization tool)


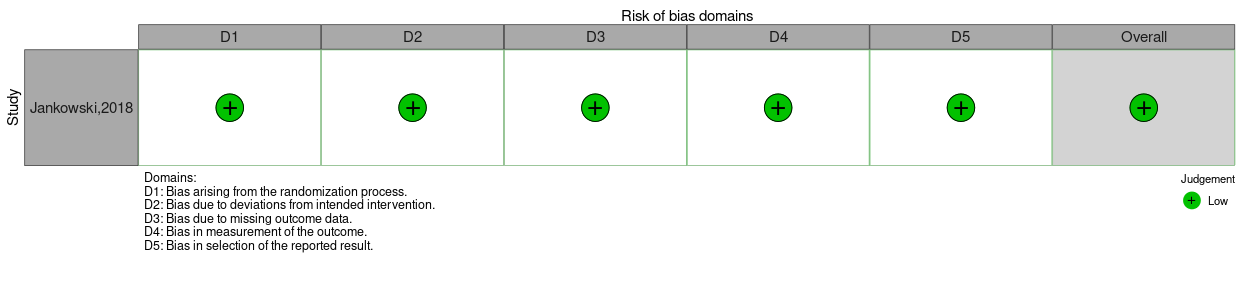


# Supplementary Figure 3 . Weighted bar plots of the distribution of risk-of-bias judgements within each bias domain of the ROB2 tool using the ROBVIS ( visualization tool)


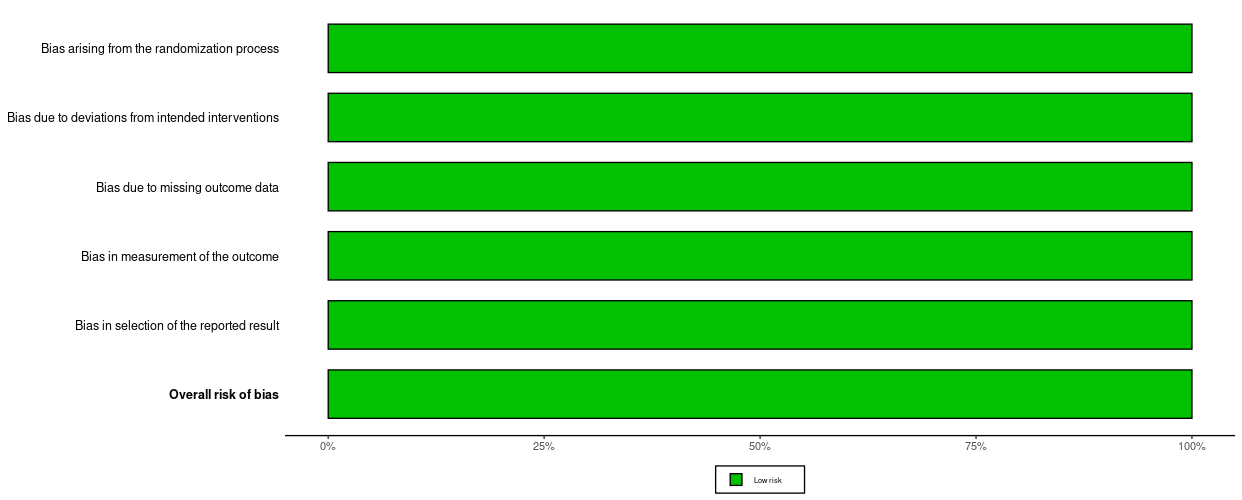


# Supplementary Table 6. GRADE summary of findings table. Use of statins, aspirin and proton pump inhibitors compared with no use in patients with Barrett’s esophagus for the outcome of high-grade dysplasia or cancer.

| **№ of studies** | **Certainty assessment** | | | | | | **Effect** | | | | **Certainty** |
| --- | --- | --- | --- | --- | --- | --- | --- | --- | --- | --- | --- |
|  | **Study design** | **Risk of bias** | **Inconsistency** | **Indirectness** | **Imprecision** | **Other considerations** | **№ of events** | **№ of individuals** | **Relative effect (95% CI)** | **Risk difference (95% CI)^a^** |  |
| Statins | | | | | | | | | | | |
| 10 | observational studies | serious^b^ | serious^c^ | not serious | not serious | Nil | 1296 | 19581 | **OR 0.53** (0.37 to 0.74) | **1.55** (0.86 to 2.08) per 1000 per year | ⨁◯◯◯ Very low |
| Aspirin | | | | | | | | | | | |
| 7 | observational studies and one RCT | Serious^d^ | not serious | not serious | serious^e^ | Nil | 380 | 9833 | **OR 0.84** (0.65 to 1.08) | **0.53** (-0.26 to 1.16) per 1000 per year | ⨁◯◯◯ Very low |
| Proton pump inhibitors | | | | | | | | | | | |
| 5 | observational studies | serious^f^ | serious^g^ | not serious | not serious | Nil | 1418 | 18627 | **OR 0.46** (0.25 to 0.86) | **1.78** (2.48 to 0.46) per 1000 year | ⨁◯◯◯ Very low |

#### GRADE Working Group grades of evidence

#### **High quality**: We are very confident that the true effect lies close to that of the estimate of the effect

#### **Moderate quality**: We are moderately confident in the effect estimate: The true effect is likely to be close to the estimate of the effect, but there is a possibility that it is substantially different

#### **Low quality**: Our confidence in the effect estimate is limited: The true effect may be substantially different from the estimate of the effect

#### Very low quality: We have very little confidence in the effect estimate: The true effect is likely to be substantially different from the estimate of effect

#### Explanations

#### Non-randomised studies considered low certainty evidence at baseline.

1. Assumed comparator risk (ACR) is 33 per 1000 per year (Desai 2012). Risk difference per 1000 = 1000 x ACR x (1-RR)
2. 3 studies at critical risk of bias, 7 studies at serious risk of bias. Downgraded 1 levels.
3. Considerable heterogeneity (I^2^ = 79.5%). Downgraded 1 level.
4. 3 studies at critical risk of bias, 3 at serious risk of bias, 1 at low risk of bias. Downgraded 1 levels.
5. 95% CI crosses the null with up to 35% reduced risk or 8% increased risk. Downgraded 1 level.
6. 3 studies at critical risk of bias, 8 at serious risk of bias. Downgraded 2 levels.
7. Considerable heterogeneity (I^2^ = 92.2%). Downgraded 1 level.

# Supplementary Figure 4. Subgroup Analysis Based on Study Design (PPI)


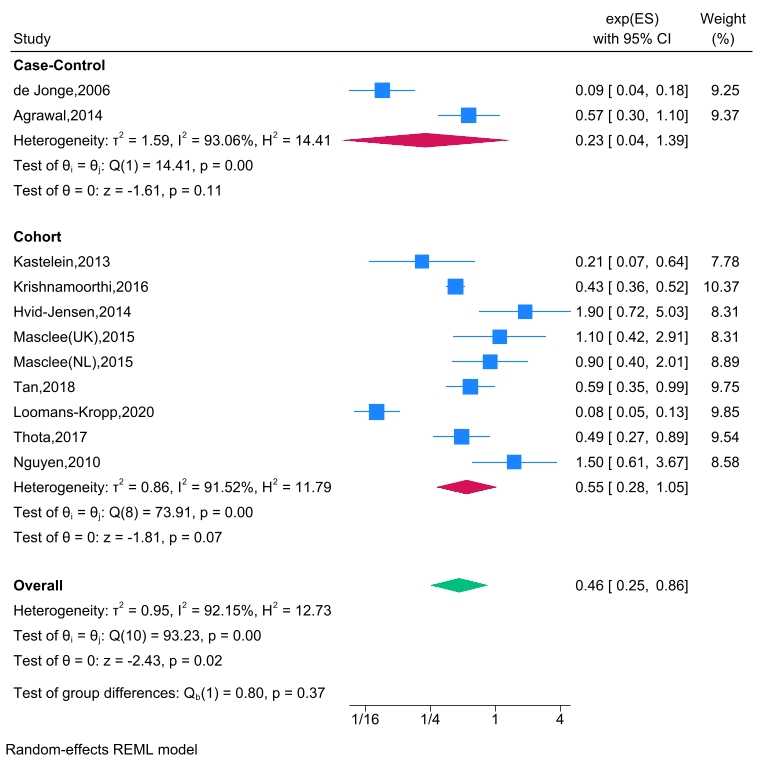


Effect size (ES) – Odds ratio (OR) with 95% confidence intervals ( CI)

# Supplementary Figure 5. Subgroup Analysis Based on Study Setting (PPI)


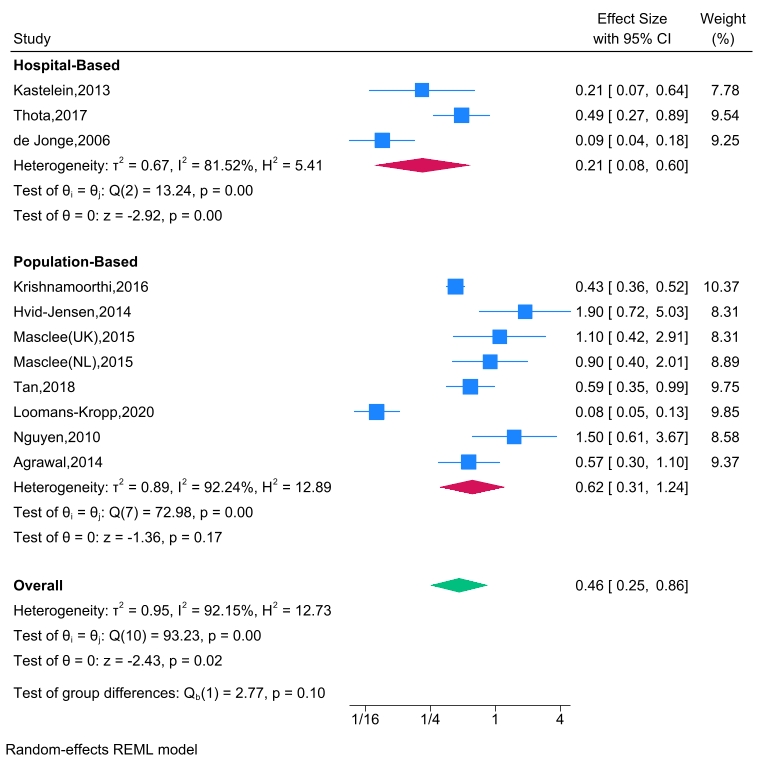


Effect size (ES) – Odds ratio (OR) with 95% confidence intervals ( CI)

# Supplementary Figure 6 . Subgroup Analysis Based on baseline dysplastic status (PPI)


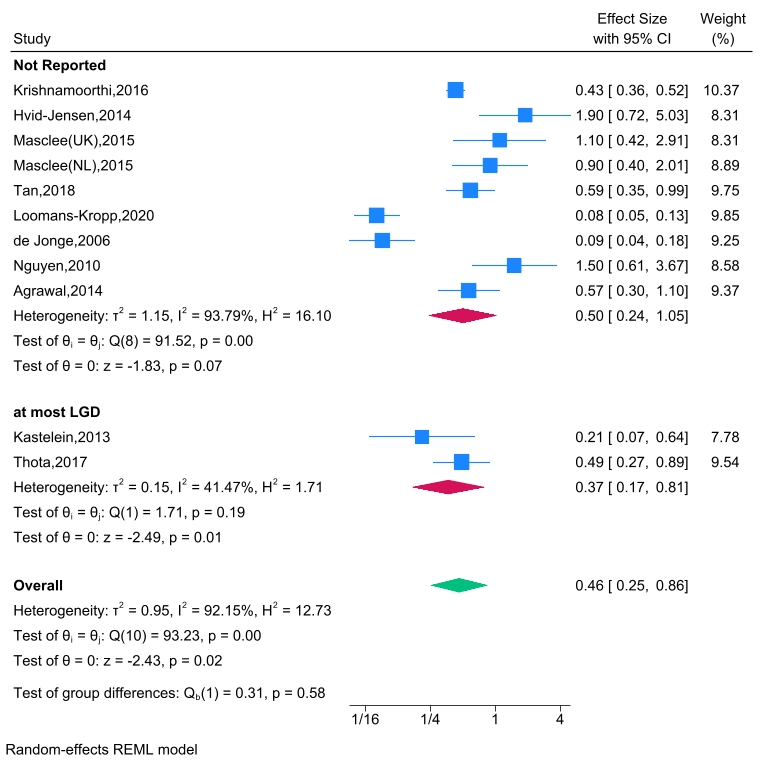


Effect size (ES) – Odds ratio (OR) with 95% confidence intervals ( CI)

Supplementary Figure 7. Subgroup Analysis Based on Study Outcome(PPI)


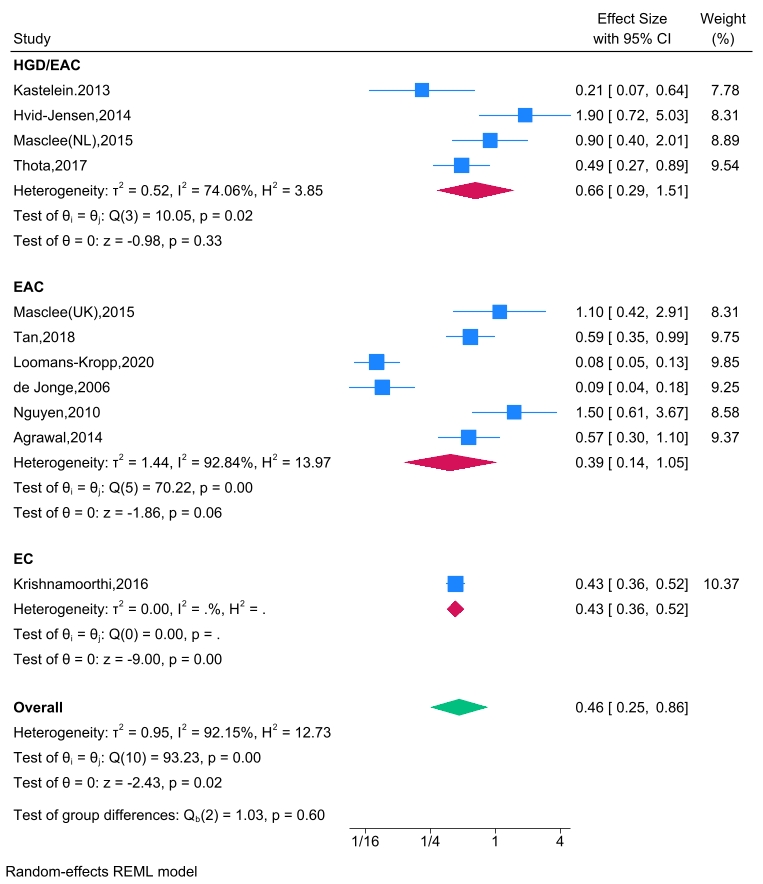


Effect size (ES) – Odds ratio (OR) with 95% confidence intervals ( CI)

# Supplementary Figure 8. Subgroup Analysis Based on exposure ascertainment(PPI)


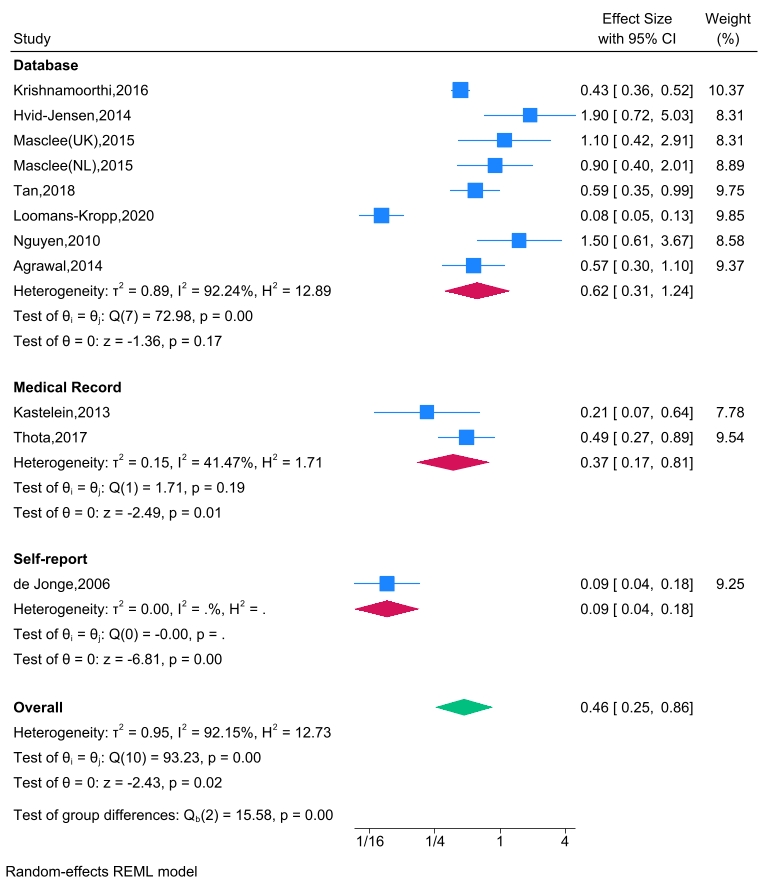


Effect size (ES) – Odds ratio (OR) with 95% confidence intervals ( CI)

# Supplementary Figure 9. Subgroup Analysis Based on risk of bias assessment(PPI)


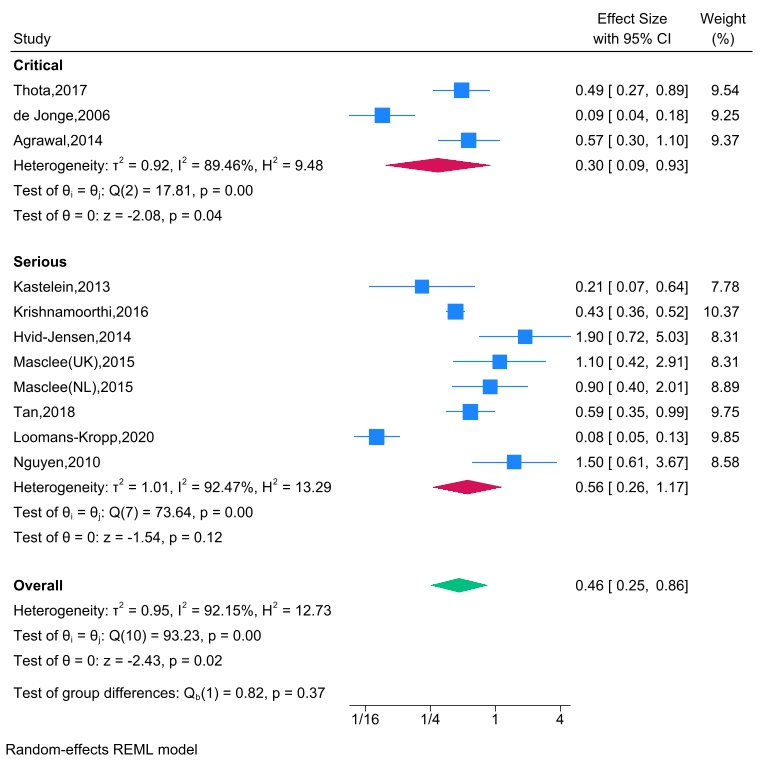


Effect size (ES) – Odds ratio (OR) with 95% confidence intervals ( CI)

# Supplementary Figure 10. Subgroup Analysis Based on definition of BE(PPI)

#
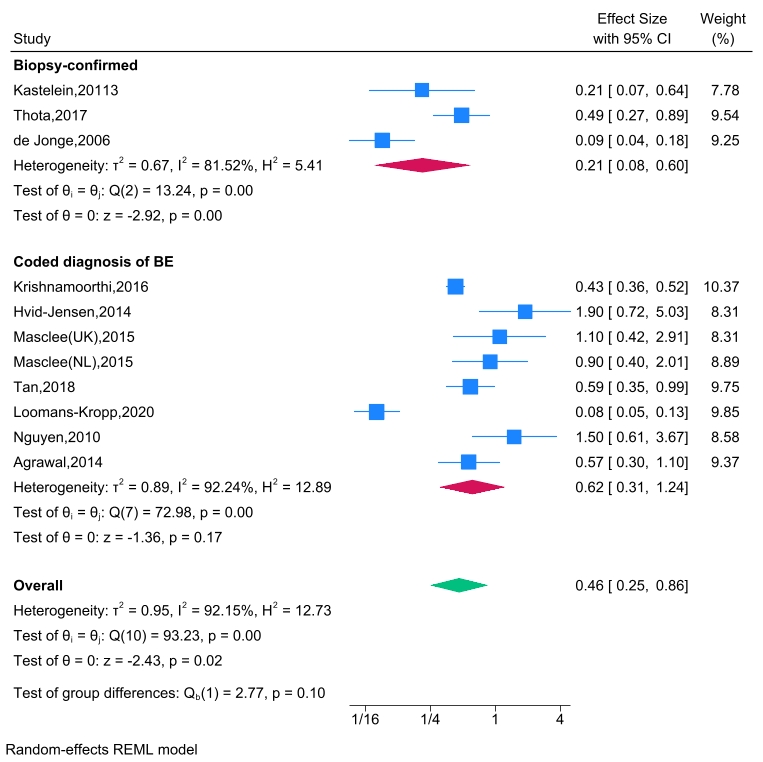


# Supplementary Figure 24. Subgroup Analysis Based on BE length (PPI)

#
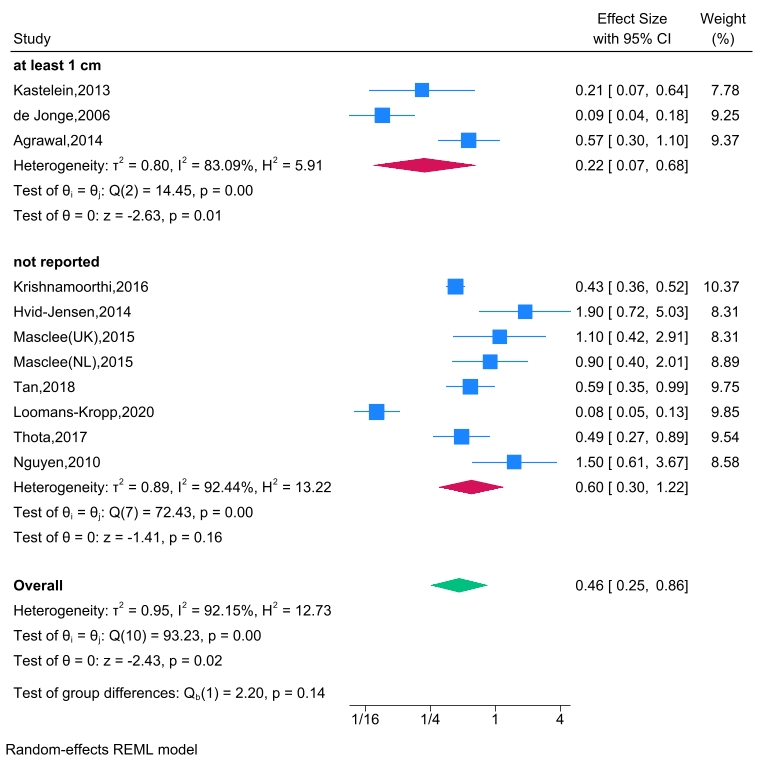


# Supplementary Figure 12. Subgroup Analysis Based on Study Design(aspirin)


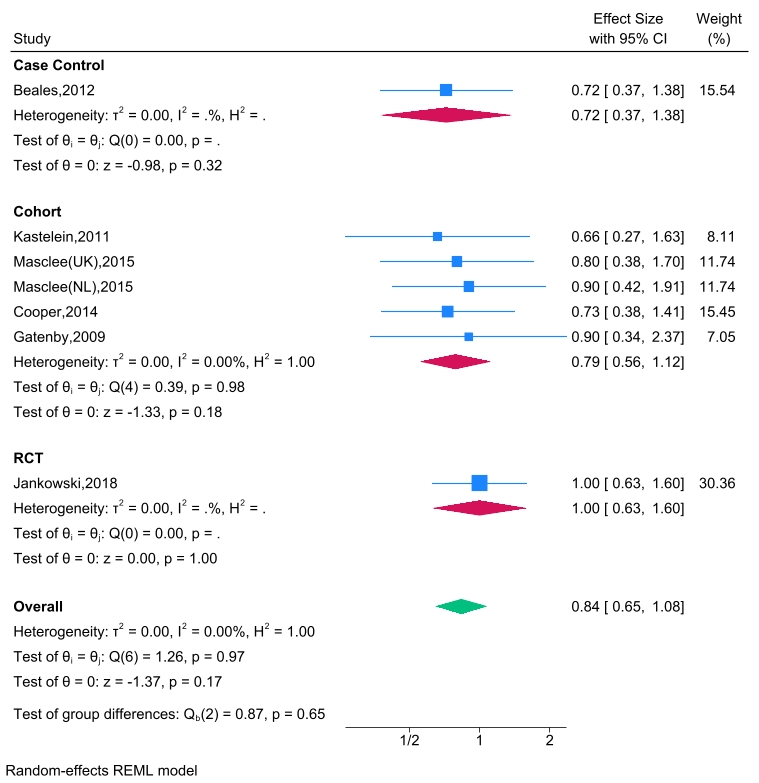


RCT = Randomised Controlled Trial

Effect size (ES) – Odds ratio (OR) with 95% confidence intervals ( CI)

# Supplementary Figure 13. Subgroup Analysis Based on study setting ( aspirin)


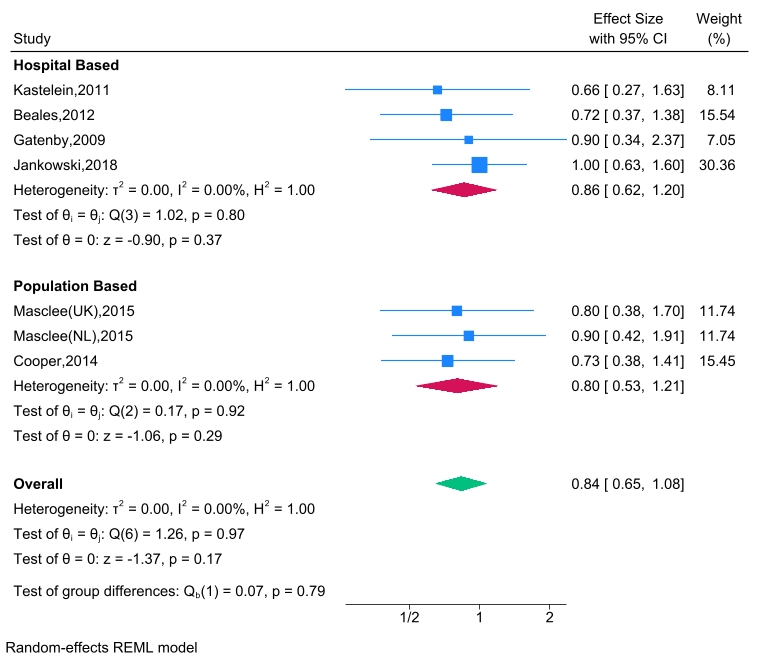


Effect size (ES) – Odds ratio (OR) with 95% confidence intervals ( CI)

# Supplementary Figure 14. Subgroup Analysis Based on Baseline Dysplastic Status(aspirin)


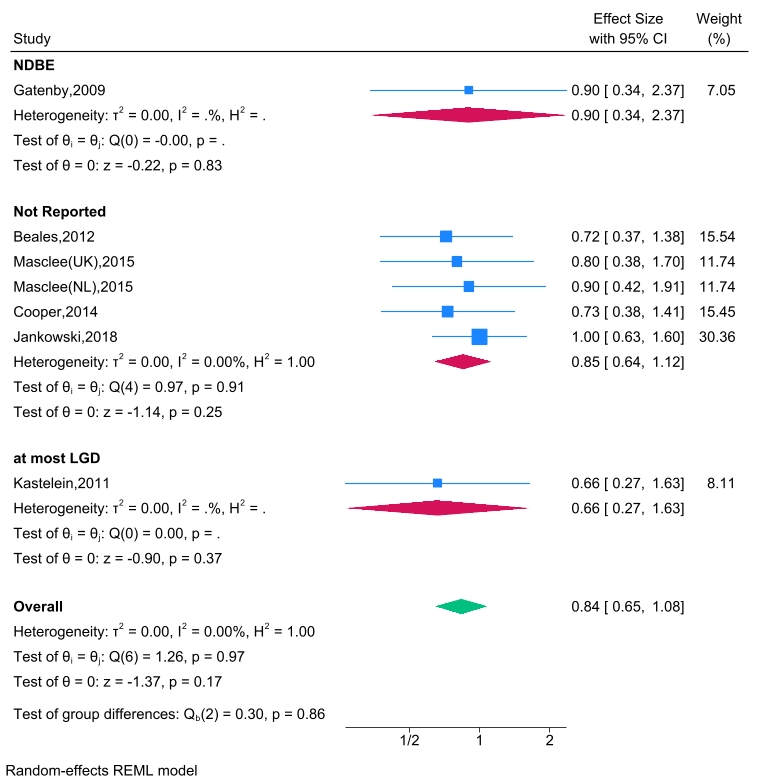


Effect size (ES) – Odds ratio (OR) with 95% confidence intervals (CI)

# Supplementary Figure 15. Subgroup Analysis Based on study outcome(aspirin)


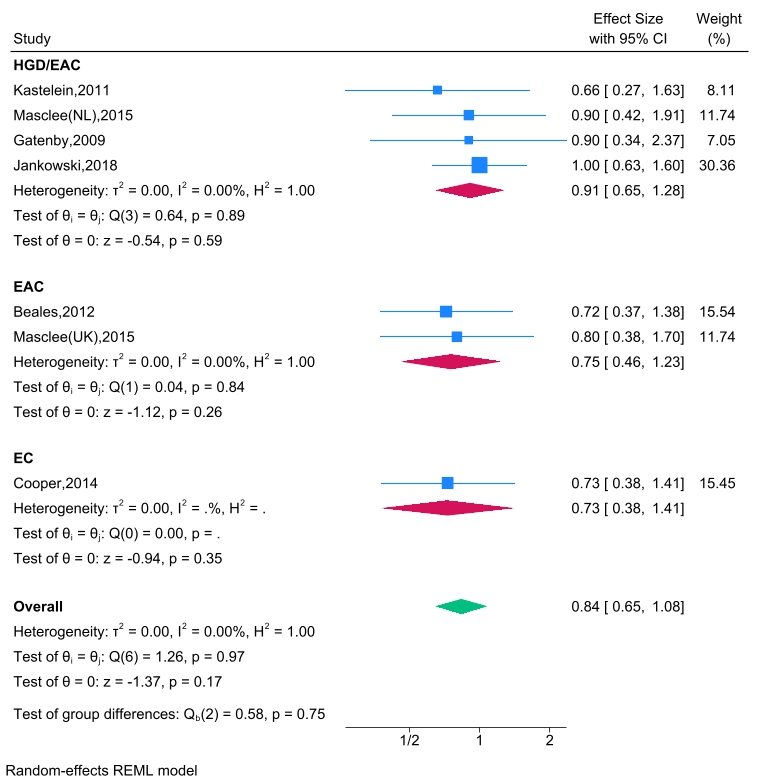


Effect size (ES) – Odds ratio (OR) with 95% confidence intervals ( CI)

# Supplementary Figure 16. Subgroup Analysis Based on exposure ascertainment (aspirin)


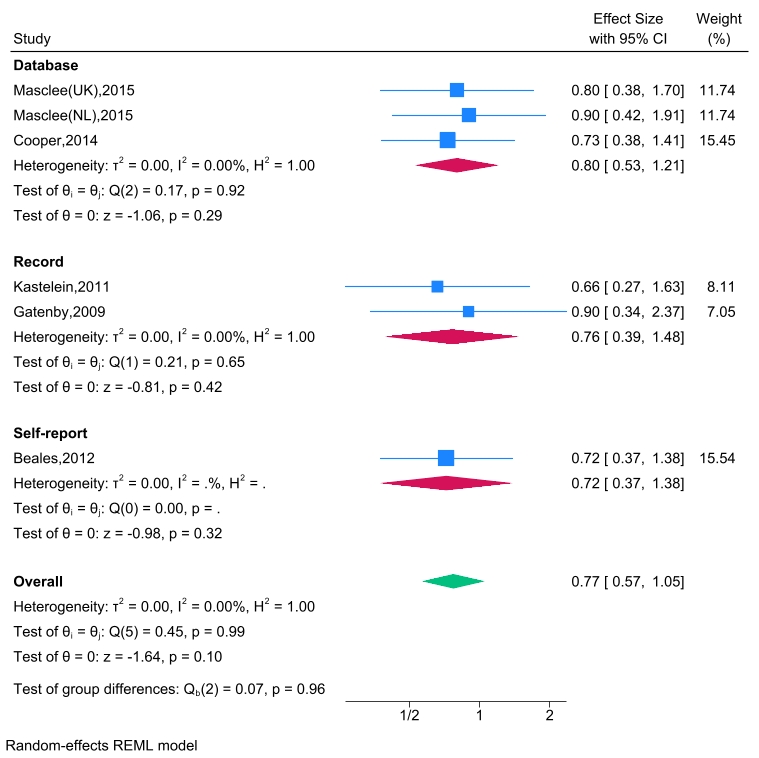


Effect size (ES) – Odds ratio (OR) with 95% confidence intervals ( CI)

# Supplementary Figure 17. Subgroup Analysis Based on risk of bias assessment (aspirin)


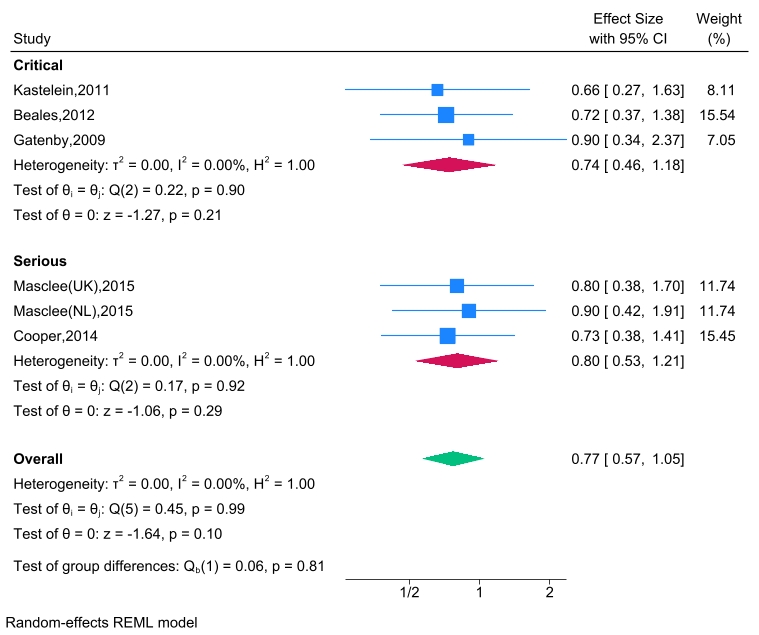


Effect size (ES) – Odds ratio (OR) with 95% confidence intervals ( CI)

# Supplementary Figure 18. Subgroup Analysis Based on BE definition (aspirin)


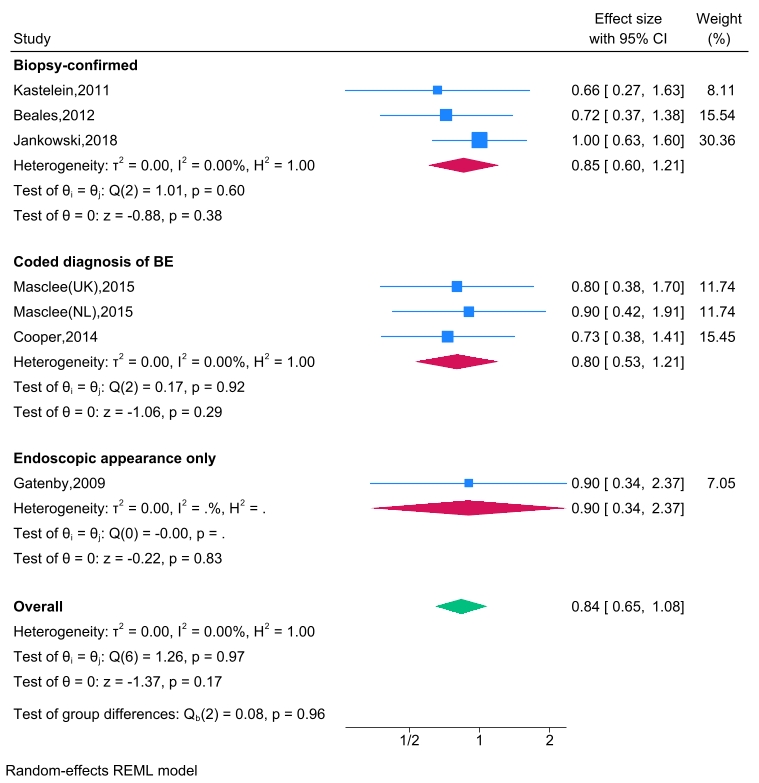


# Supplementary Figure 19. Subgroup Analysis Based on BE length (aspirin)

#
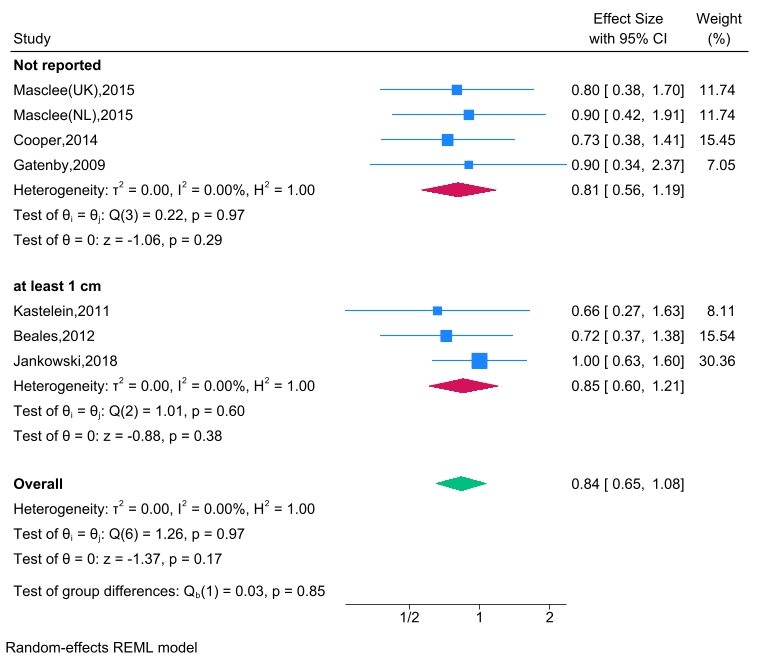


# Supplementary Figure 20. Subgroup Analysis Based on study design (statin)


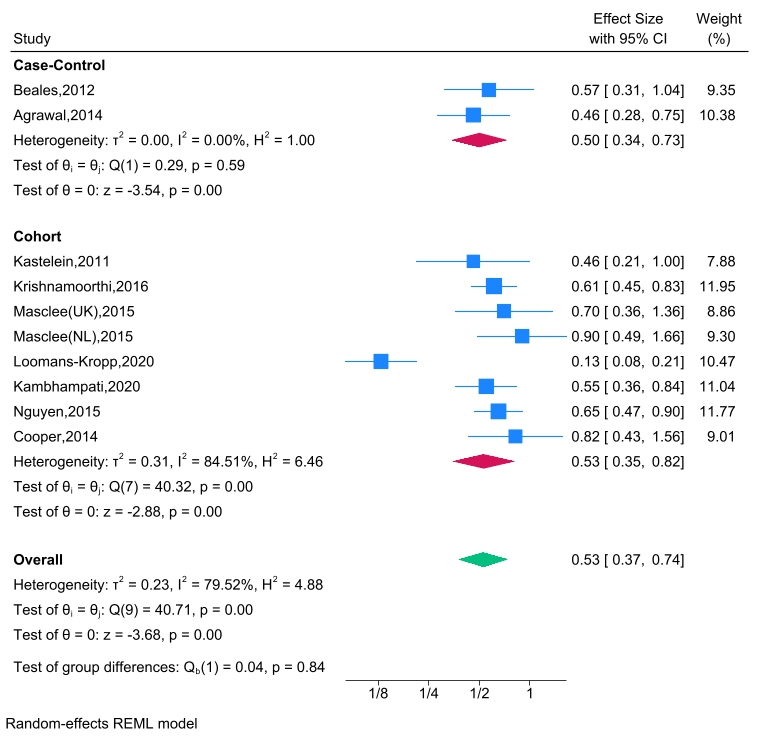


Effect size (ES) – Odds ratio (OR) with 95% confidence intervals ( CI)

# Supplementary Figure 21. Subgroup Analysis Based on Study Setting (statin)


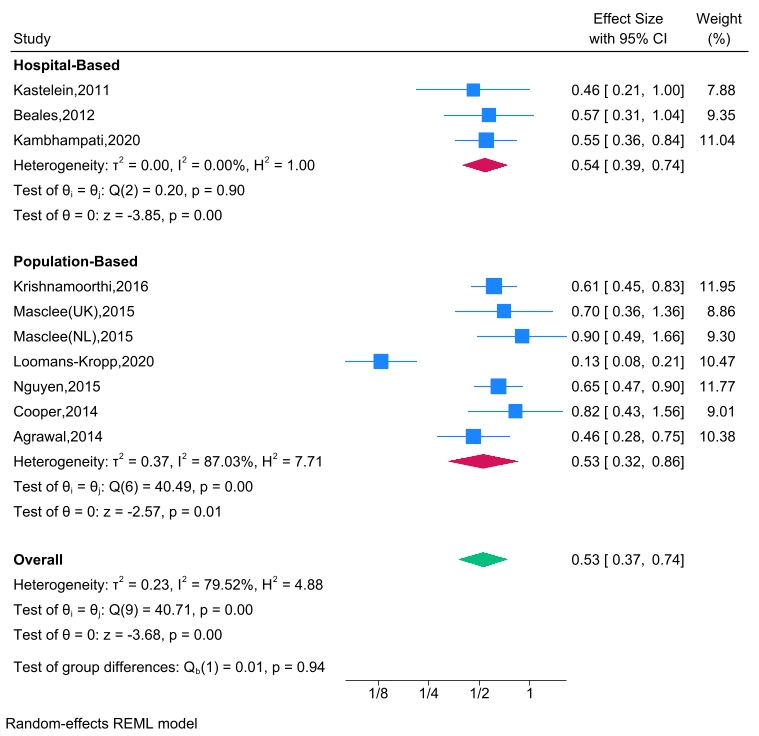


Effect size (ES) – Odds ratio (OR) with 95% confidence intervals ( CI)

# Supplementary Figure 22 . Subgroup Analysis Based on Baseline Dysplastic Status (statin)


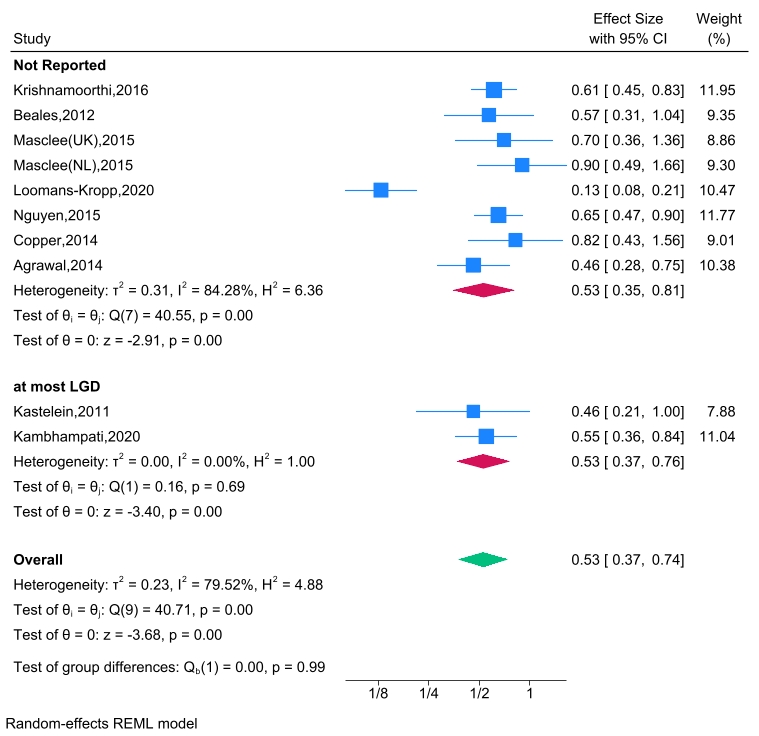


Effect size (ES) – Odds ratio (OR) with 95% confidence intervals ( CI)

# Supplementary Figure 23. Subgroup Analysis Based on study outcome (Statin)


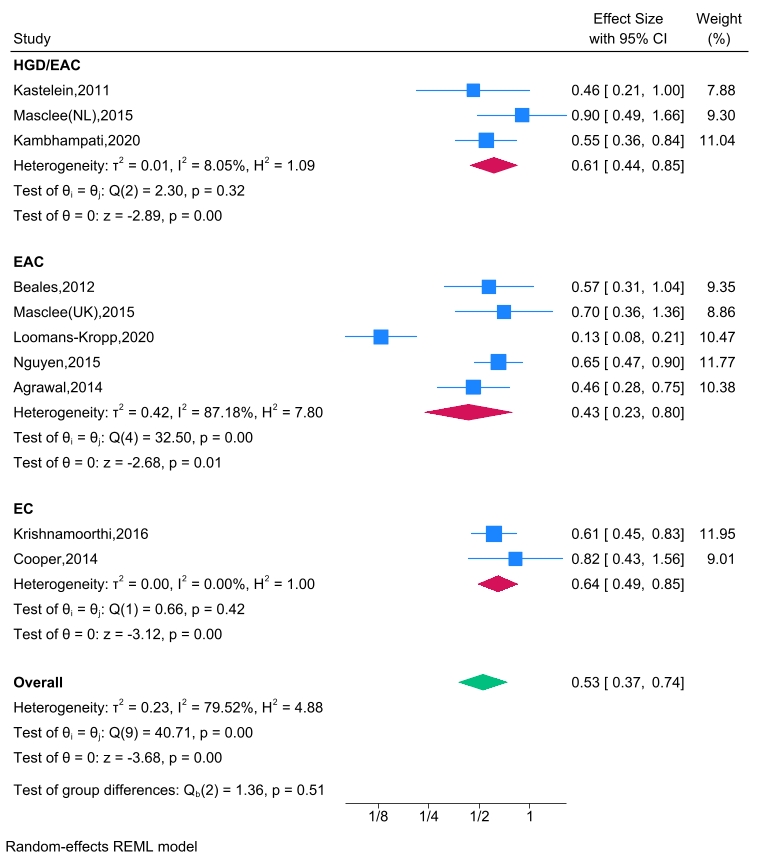


Effect size (ES) – Odds ratio (OR) with 95% confidence intervals ( CI)

# Supplementary Figure 24. Subgroup Analysis Based on exposure ascertainment (statin)


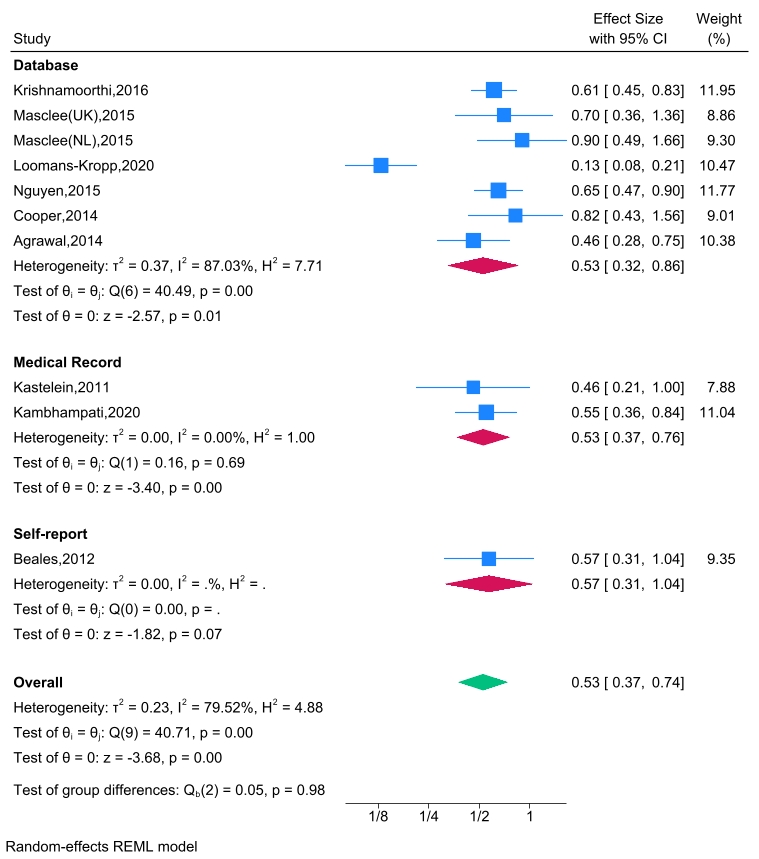


Effect size (ES) – Odds ratio (OR) with 95% confidence intervals ( CI)

# Supplementary Figure 25. Subgroup Analysis Based on risk of bias assessment (statin)


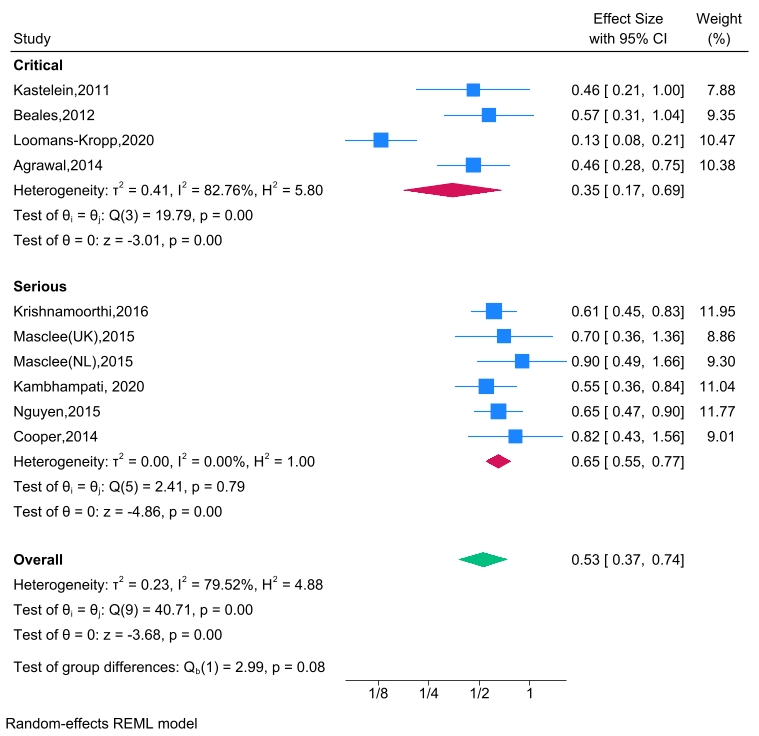


Effect size (ES) – Odds ratio (OR) with 95% confidence intervals ( CI)

# Supplementary Figure 26. Subgroup Analysis Based on BE definition (statin)


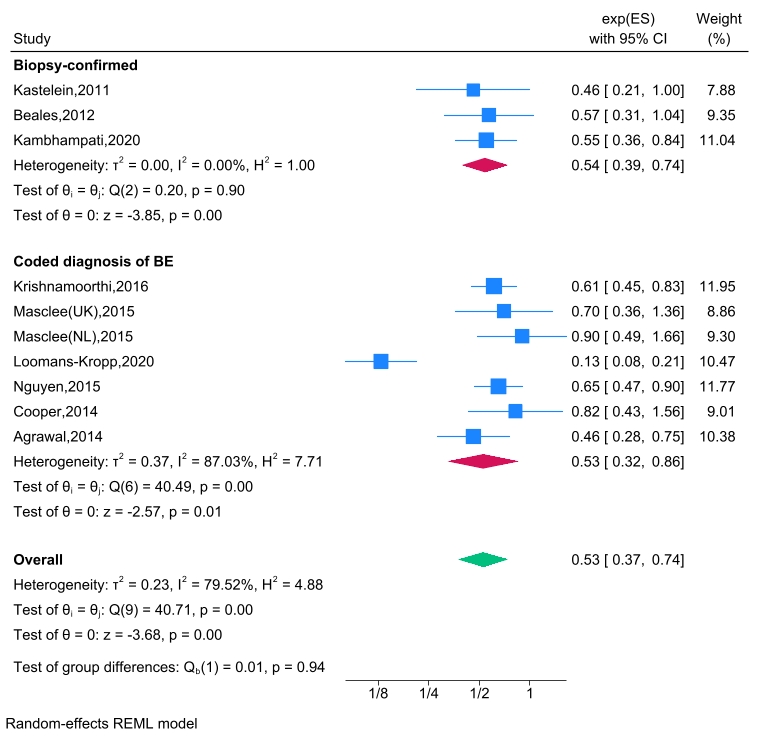


# Supplementary Figure 27. Subgroup Analysis Based on BE length (statin)


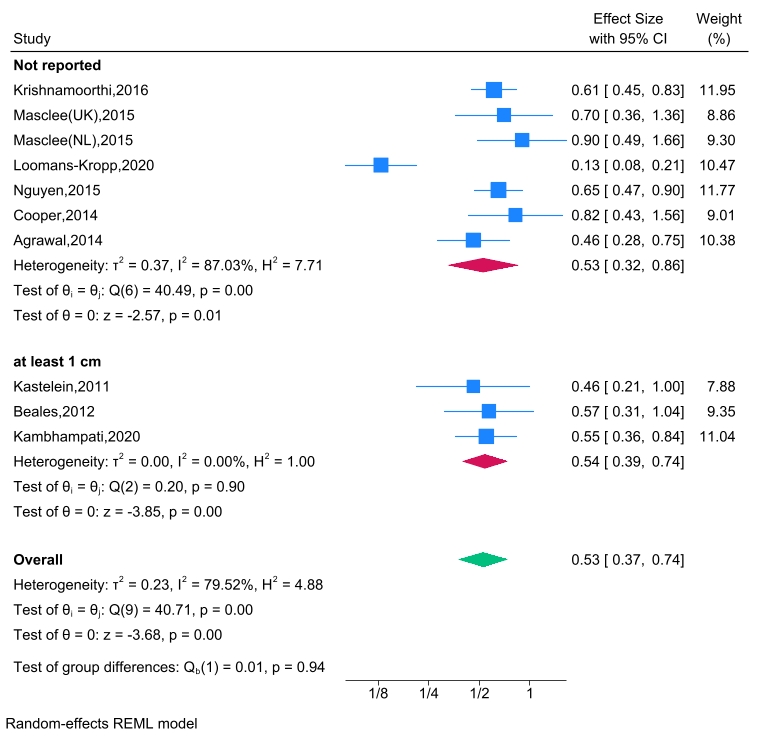


# Supplementary figure 28. Funnel plot to assess publication bias


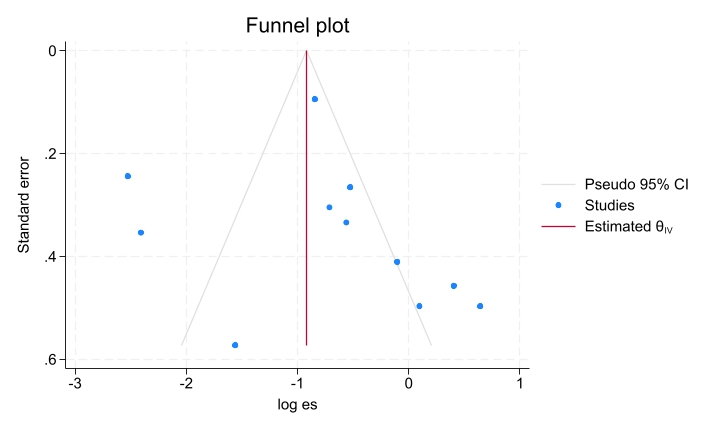


Egger’s regression test: p=0.39

# Supplementary figure 29: Directed Acyclic Graph (DAG): the assumed causal relationships between PPIs and malignant progression of Barrett’s esophagus.


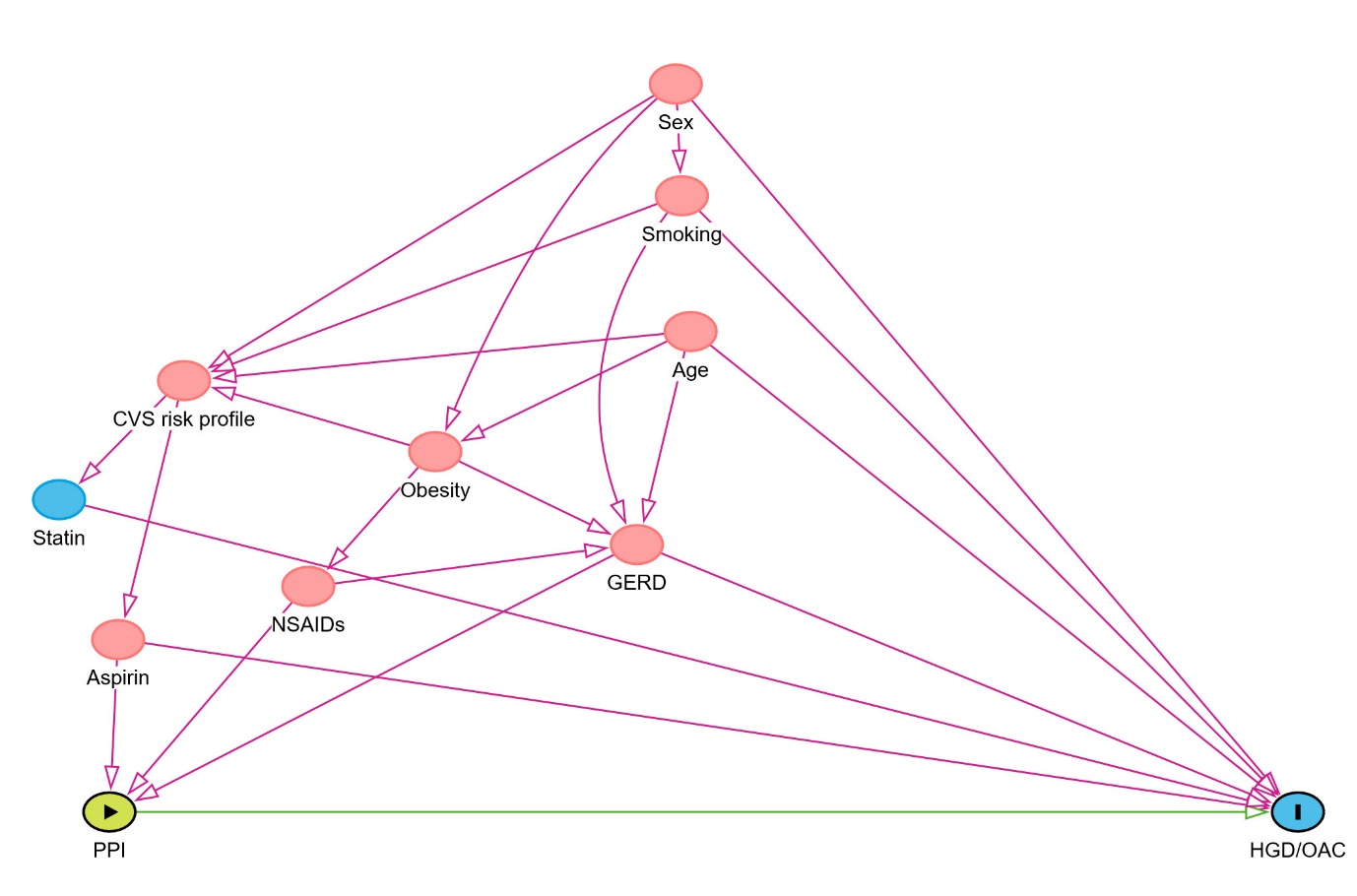


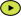
exposure


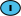
outcome


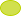
ancestor of exposure


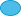
ancestor of outcome


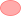
ancestor of exposure *and* outcome


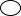
adjusted variable


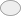
unobserved (latent)


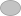
other variable


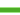
causal path


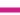
biasing path

Minimal sufficient adjustment sets for estimating the total effect of PPI on HGD/EAC:

- Age, Aspirin, CVS risk profile, GERD, Sex, Smoking
- Age, Aspirin, GERD, Obesity, Smoking
- Age, Aspirin, GERD, Sex, Smoking, Statin
- Aspirin, GERD, NSAIDs

# Supplementary figure 30: Directed Acyclic Graph (DAG): the assumed causal relationships between aspirin and malignant progression of Barrett’s esophagus.

#
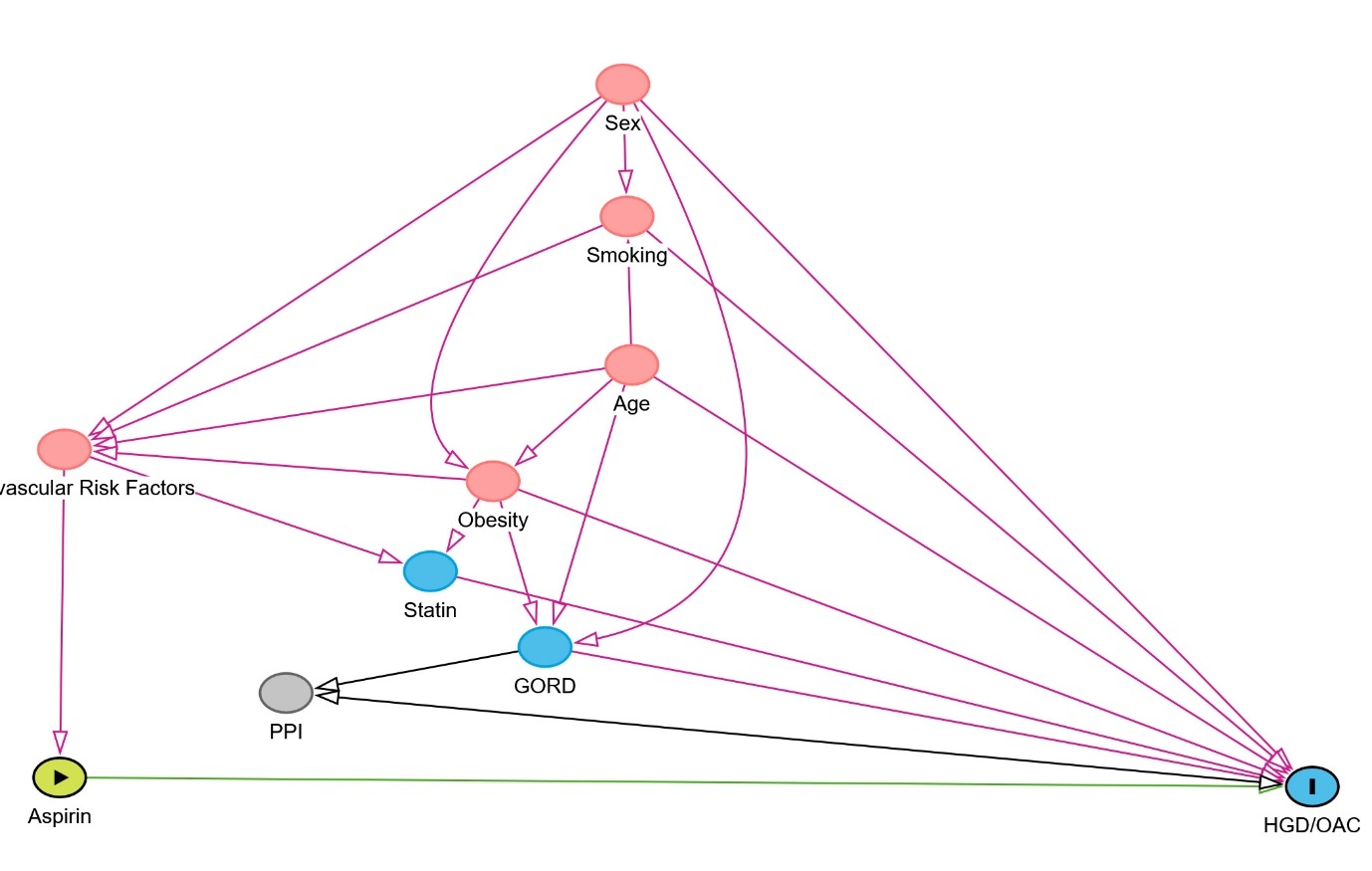


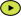
exposure


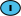
outcome


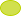
ancestor of exposure


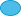
ancestor of outcome


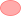
ancestor of exposure *and* outcome


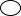
adjusted variable


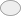
unobserved (latent)


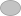
other variable


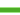
causal path

biasing path


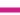


Minimal sufficient adjustment sets for estimating the total effect of aspirin on HGD/EAC:

- Age, Obesity, Sex, Smoking, Statin
- Cardiovascular Risk Factor

# Supplementary figure 31: Directed Acyclic Graph (DAG): the assumed causal relationships between statins and malignant progression of Barrett’s esophagus.


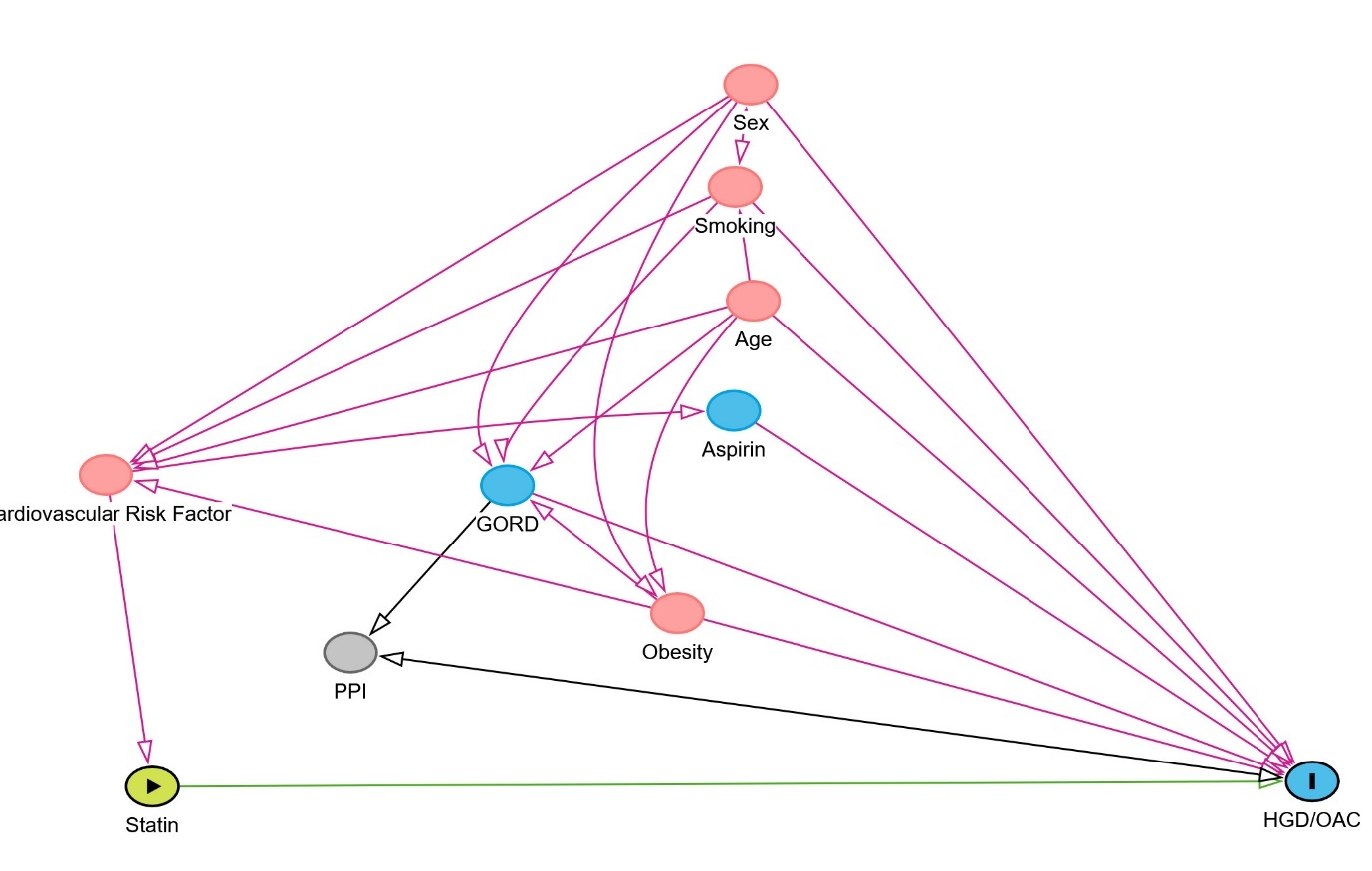


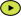
exposure


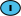
outcome


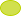
ancestor of exposure


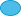
ancestor of outcome


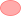
ancestor of exposure *and* outcome


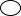
adjusted variable


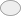
unobserved (latent)


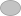
other variable


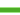
causal path


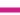
biasing path

Minimal sufficient adjustment sets for estimating the total effect of statin on HGD/EAC:

- Age, Aspirin, Obesity, Sex, Smoking
- Cardiovascular Risk Factor

References

1. Beales IL, Dearman L, Vardi I, et al. Reduced Risk of Barrett's Esophagus in Statin Users: Case-Control Study and Meta-Analysis. Dig Dis Sci 2016;61:238-46.

2. Brown CS, Lapin B, Wang C, et al. Reflux control is important in the management of Barrett's Esophagus: results from a retrospective 1,830 patient cohort. Surg Endosc 2015;29:3528-34.

3. Beales IL, Vardi I, Dearman L, et al. Statin use is associated with a reduction in the incidence of esophageal adenocarcinoma: a case control study. Dis Esophagus 2013;26:838-46.

4. Kantor ED, Onstad L, Blount PL, et al. Use of statin medications and risk of esophageal adenocarcinoma in persons with Barrett's esophagus. Cancer Epidemiol Biomarkers Prev 2012;21:456-61.

5. Hillman LC, Chiragakis L, Shadbolt B, et al. Effect of proton pump inhibitors on markers of risk for high-grade dysplasia and oesophageal cancer in Barrett's oesophagus. Aliment Pharmacol Ther 2008;27:321-6.

6. Vaughan TL, Dong LM, Blount PL, et al. Non-steroidal anti-inflammatory drugs and risk of neoplastic progression in Barrett's oesophagus: a prospective study. Lancet Oncol 2005;6:945-52.

7. Hillman LC, Chiragakis L, Shadbolt B, et al. Proton-pump inhibitor therapy and the development of dysplasia in patients with Barrett's oesophagus. Med J Aust 2004;180:387-91.

8. El-Serag HB, Aguirre TV, Davis S, et al. Proton Pump Inhibitors Are Associated with Reduced Incidence of Dysplasia in Barrett's Esophagus. Official journal of the American College of Gastroenterology | ACG 2004;99:1877-1883.

9. Gatenby PA, Ramus JR, Caygill CP, et al. Treatment modality and risk of development of dysplasia and adenocarcinoma in columnar-lined esophagus. Dis Esophagus 2009;22:133-42.

10. Nguyen DM, El-Serag HB, Henderson L, et al. Medication usage and the risk of neoplasia in patients with Barrett's esophagus. Clin Gastroenterol Hepatol 2009;7:1299-304.

11. Cooper BT, Chapman W, Neumann CS, et al. Continuous treatment of Barrett's oesophagus patients with proton pump inhibitors up to 13 years: observations on regression and cancer incidence. Aliment Pharmacol Ther 2006;23:727-33.

12. Tsibouris P, Hendrickse MT, Isaacs PE. Daily use of non-steroidal anti-inflammatory drugs is less frequent in patients with Barrett's oesophagus who develop an oesophageal adenocarcinoma. Aliment Pharmacol Ther 2004;20:645-55.

13. Galipeau PC, Li X, Blount PL, et al. NSAIDs modulate CDKN2A, TP53, and DNA content risk for progression to esophageal adenocarcinoma. PLoS Med 2007;4:e67.

14. Ortiz A, Haro LFM, Parrilla P, et al. Conservative treatment versus antireflux surgery in Barrett's oesophagus: Long-term results of a prospective study. British Journal of Surgery 1996;83:274-278.

15. Babic Z, Bogdanovic Z, Dorosulic Z, et al. One year treatment of Barrett<b>’</b>s oesophagus with proton pump inhibitors (a multi-center study). Acta Clinica Belgica 2015, 70(6), 408–413. https://doi.org/10.1179/2295333715Y.0000000050

# 
